# Intestinal Osteopontin Protects From Alcohol-induced Liver Injury by Preserving the Gut Microbiome and the Intestinal Barrier Function

**DOI:** 10.1016/j.jcmgh.2022.06.012

**Published:** 2022-07-08

**Authors:** Sukanta Das, Zhuolun Song, Hui Han, Xiaodong Ge, Romain Desert, Dipti Athavale, Sai Santosh Babu Komakula, Fernando Magdaleno, Wei Chen, Daniel Lantvit, Grace Guzman, Natalia Nieto

**Affiliations:** 1 Department of Pathology, University of Illinois at Chicago, Chicago, Illinois; 2 Department of Medicine, Division of Gastroenterology and Hepatology, University of Illinois at Chicago, Chicago, Illinois; 3 Research Biologist, Research & Development Service, Jesse Brown Veterans Affairs Medical Center, Chicago, Illinois

**Keywords:** Antimicrobial Peptides, Aryl Hydrocarbon Receptor, Gut-liver Axis, Short-chain Fatty Acids, Tryptophan, Ahr, aryl hydrocarbon receptor, ALD, alcohol-associated liver disease, ALT, alanine aminotransferase, AMPs, antimicrobial peptides, FC, fold change, FITC-dextran, fluorescein isothiocyanate-dextran, FM, fecal microbiome, FMT, fecal microbiome transplant, H&E, hematoxylin and eosin, IECs, intestinal epithelial cells, Il1β, interleukin-1β, IM, intestinal microbiome, Jam4, junctional adhesion molecule 4, JamA, junctional adhesion molecule A, KCs, Kupffer cells, LC/MS, liquid chromatography–mass spectrometry, LDC, Lieber-DeCarli, LPS, lipopolysaccharide, MFs, macrophages, mOPN, milk osteopontin, OPN, osteopontin, Reg, Regenerating islet-derived protein, SCFAs, short-chain fatty acids, TGs, triglycerides, TJ, tight-junction, Tnfα, tumor necrosis factor-α, Trp, tryptophan, WT, wild-type

## Abstract

**Background & Aims:**

The gut-liver axis plays a key role in the pathogenesis of alcohol-associated liver disease (ALD). We demonstrated that *Opn*^-/-^ develop worse ALD than wild-type (WT) mice; however, the role of intestinal osteopontin (OPN) in ALD remains unknown. We hypothesized that overexpression of OPN in intestinal epithelial cells (IECs) could ameliorate ALD by preserving the gut microbiome and the intestinal barrier function.

**Methods:**

*Opn*^KI IEC^, *Opn*^ΔIEC^, and WT mice were fed control or ethanol Lieber-DeCarli diet for 6 weeks.

**Results:**

*Opn*^KI IEC^ but not *Opn*^ΔIEC^ mice showed improved intestinal barrier function and protection from ALD. There were less pathogenic and more beneficial bacteria in ethanol-fed *Opn*^KI IEC^ than in WT mice*.* Fecal microbiome transplant (FMT) from *Opn*^KI IEC^ to WT mice protected from ALD. FMT from ethanol-fed WT to *Opn*^KI IEC^ mice failed to induce ALD. Antimicrobial peptides, Il33, pSTAT3, aryl hydrocarbon receptor (Ahr), and tight-junction protein expression were higher in IECs from jejunum of ethanol-fed *Opn*^KI IEC^ than of WT mice. Ethanol-fed *Opn*^KI IEC^ showed more tryptophan metabolites and short-chain fatty acids in portal serum than WT mice. FMT from *Opn*^KI IEC^ to WT mice enhanced IECs Ahr and tight-junction protein expression. Oral administration of milk OPN replicated the protective effect of *Opn*^KI IEC^ mice in ALD.

**Conclusion:**

Overexpression of OPN in IECs or administration of milk OPN maintain the intestinal microbiome by intestinal antimicrobial peptides. The increase in tryptophan metabolites and short-chain fatty acids signaling through the Ahr in IECs, preserve the intestinal barrier function and protect from ALD.

## Graphical abstract

**Figure:**
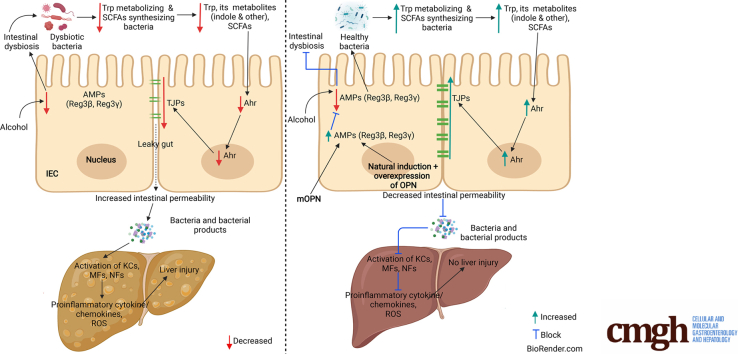

**Summary**

Osteopontin protects from alcohol-associated liver disease by regulating the intestinal microbiome, which restores tryptophan metabolites and short-chain fatty acids signaling through aryl hydrocarbon receptor in intestinal epithelial cells. This preserves the integrity of the intestinal epithelial barrier and prevents bacterial translocation from the gut to the liver.

Chronic alcohol consumption is a major cause of liver-related morbidity and mortality worldwide.^1^^,^^2^ The gut-liver axis is a key player in alcohol-associated liver disease (ALD).^3^ Alcohol and its metabolite acetaldehyde, disrupt the intestinal epithelial barrier.^4^ Moreover, alcohol lowers production of antimicrobial peptides (AMPs),^5^^,^^6^ causes intestinal dysbiosis – or changes in the composition and abundance of bacterial species – and bacterial overgrowth.^5^ Intestinal dysbiosis leads to global changes in intestinal metabolites, including branched-chain amino acids, such as tryptophan (Trp), short-chain fatty acids (SCFAs), and bile acids.^7^, ^8^, ^9^ As a result, there is disruption of the intestinal barrier, leaky gut, and translocation of bacteria and bacterial products from the intestinal lumen to the portal vein, which upon reaching the liver, activate Kupffer cells (KCs) and infiltrating macrophages (MFs), to trigger liver injury.^7^^,^^10^

Osteopontin (OPN) (encoded by the *Spp1* gene) is a soluble cytokine and a matrix-associated protein present in most tissues and body fluids.^11^^,^^12^ Serum and hepatic OPN increase in patients with alcoholic hepatitis and in animal models of ALD.^13^^,^^14^ Importantly, OPN is expressed in intestinal epithelial cells (IECs)^15^ and maintains tight-junction (TJ) protein complexes, enabling occludin to localize to the TJs to be phosphorylated.^15^ Furthermore, OPN maintains homeostasis of intestinal commensal bacteria by preserving TCRγδ^+^ intraepithelial lymphocytes,^16^ and acts as an opsonin to induce phagocytosis of bacteria.^17^

Previous work from our laboratory demonstrated that overexpression of OPN in hepatocytes or treatment with milk osteopontin (mOPN) protected from ALD by blocking the gut-derived lipopolysaccharide (LPS) effects in the liver.^2^ However, the role of IEC-derived OPN in ALD remains to be determined. We hypothesized that increasing OPN expression in IECs could preserve the gut-liver axis and protect from ALD. Thus, the aims of this work were, first, to compare the hepatoprotective effects of natural induction (due to alcohol) versus natural induction plus overexpression OPN in IECs; second, to analyze if enhanced expression of OPN in IECs could preserve the gut microbiome and the intestinal epithelial barrier function to protect from ALD; and third, to evaluate the therapeutic potential of oral administration of mOPN to maintain the gut microbial homeostasis under alcohol consumption.

## Results

### Ethanol Increases the Expression of OPN in IECs, Which Protects From ALD

**Figure 1:**
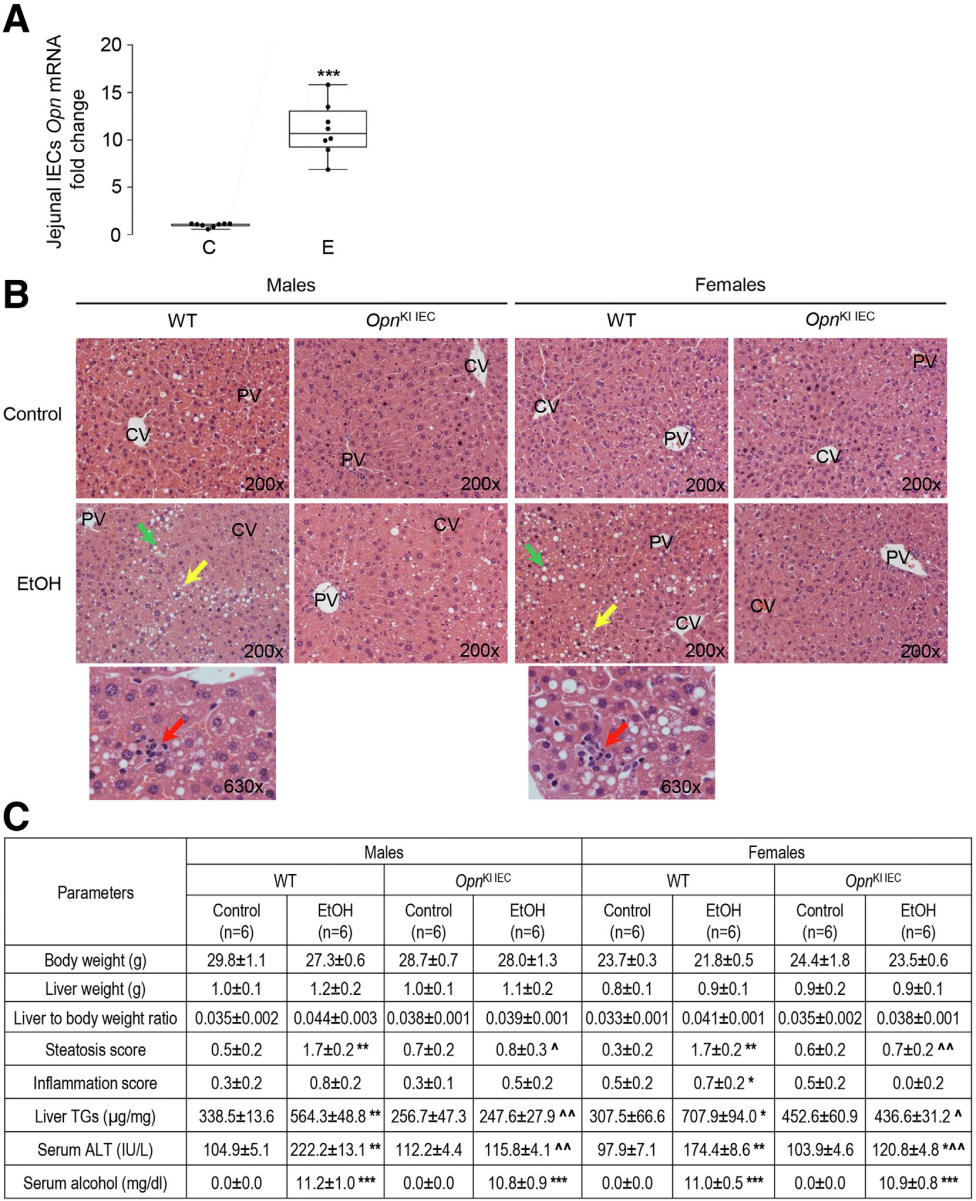

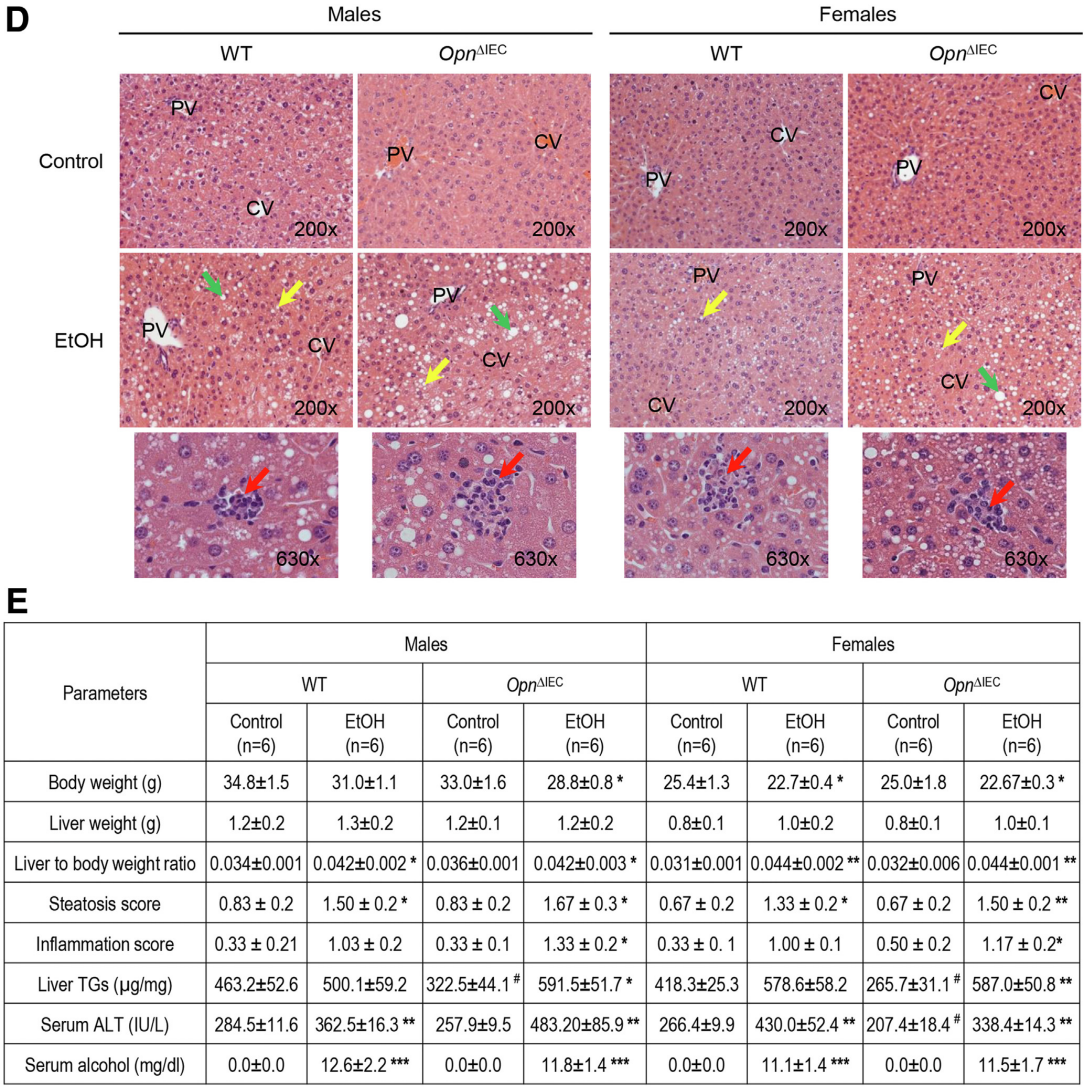
**Ethanol increases theOPNexpressionin IECs, which protects from ALD.** WT mice were fed control or ethanol diet for 6 weeks to provoke ALD. *Opn* mRNA expression was measured in IECs isolated from jejunum. Data were normalized with *Gapdh* as housekeeping gene and fold change (FC) was calculated against control. n = 8 (4 males + 4 females)/group; **∗∗∗***P* < .001 vs control (*A*). WT and *Opn*^KI IEC^ mice were fed control or ethanol diet for 6 weeks to provoke ALD. Liver H&E staining (*green arrows*, macrovesicular steatosis; *yellow arrows*, microvesicular steatosis; *red arrows*, inflammatory foci; CV, central vein; PV, portal vein) (*B*). Body weight, liver weight, liver to body weight ratio, pathology scores, liver TGs, serum ALT activity, and serum alcohol levels. n = 6/group, data are expressed as mean ± standard error of the mean. **∗***P* < .05; **∗∗***P* < .01; and **∗∗∗***P* < .001 vs control; **ˆ***P* < .05 and **ˆˆ***P* < .01 vs WT ethanol (*C*). WT and *Opn*^ΔIEC^ mice were fed control or ethanol diet for 6 weeks to provoke ALD. Liver H&E staining (*green arrows*, macrovesicular steatosis; *yellow arrows*, microvesicular steatosis; *red arrows*, inflammatory foci; CV, central vein; PV, portal vein) (*D*). Body weight, liver weight, liver to body weight ratio, pathology scores, liver TGs, serum ALT activity, and serum alcohol levels. n = 6/group, data are expressed as mean ± standard error of the mean. **∗***P* < .05; **∗∗***P* < .01; and **∗∗∗***P* < .001 vs control; ^#^*P* < .05 vs WT control. C, Control diet; E, ethanol diet (*E*).

IECs express OPN,^15^ and we previously demonstrated that ethanol drinking enhances the expression in mouse IECs.^1^ To further confirm this, we analyzed *Opn* mRNA expression in IECs of jejunum from wild-type (WT) mice fed control or ethanol Lieber-DeCarli (LDC) diet. Ethanol induced *Opn* mRNA expression in IECs (Figure 1, *A*). To evaluate the role of increased expression of OPN in IECs in ALD, male and female *Opn*^KI IEC^ and WT mice were fed control or ethanol diet for 6 weeks. Hematoxylin and eosin (H&E) staining of formalin-fixed paraffin-embedded liver sections (Figure 1, *B*), the steatosis and inflammation scores, liver triglycerides (TGs) and serum alanine aminotransferase (ALT) activity (Figure 1, *C*) showed less ethanol-induced liver injury in *Opn*^KI IEC^ than in WT mice. To determine if ablating *Opn* in IECs could worsen ALD, male and female *Opn*^ΔIEC^ and WT mice were fed control or ethanol diet for 6 weeks. The H&E staining (Figure 1, *D*), the steatosis and inflammation scores, liver TGs and serum ALT activity (Figure 1, *E*) revealed increased liver injury in ethanol-fed *Opn*^ΔIEC^ compared with WT mice. The duodenum, jejunum, ileum, and colon from these mice did not show structural changes after ethanol feeding (not shown). These findings indicate that increasing OPN expression in IECs protects from ALD.

### Increased Expression of OPN in IECs Preserves Gut Permeability

**Figure 2:**
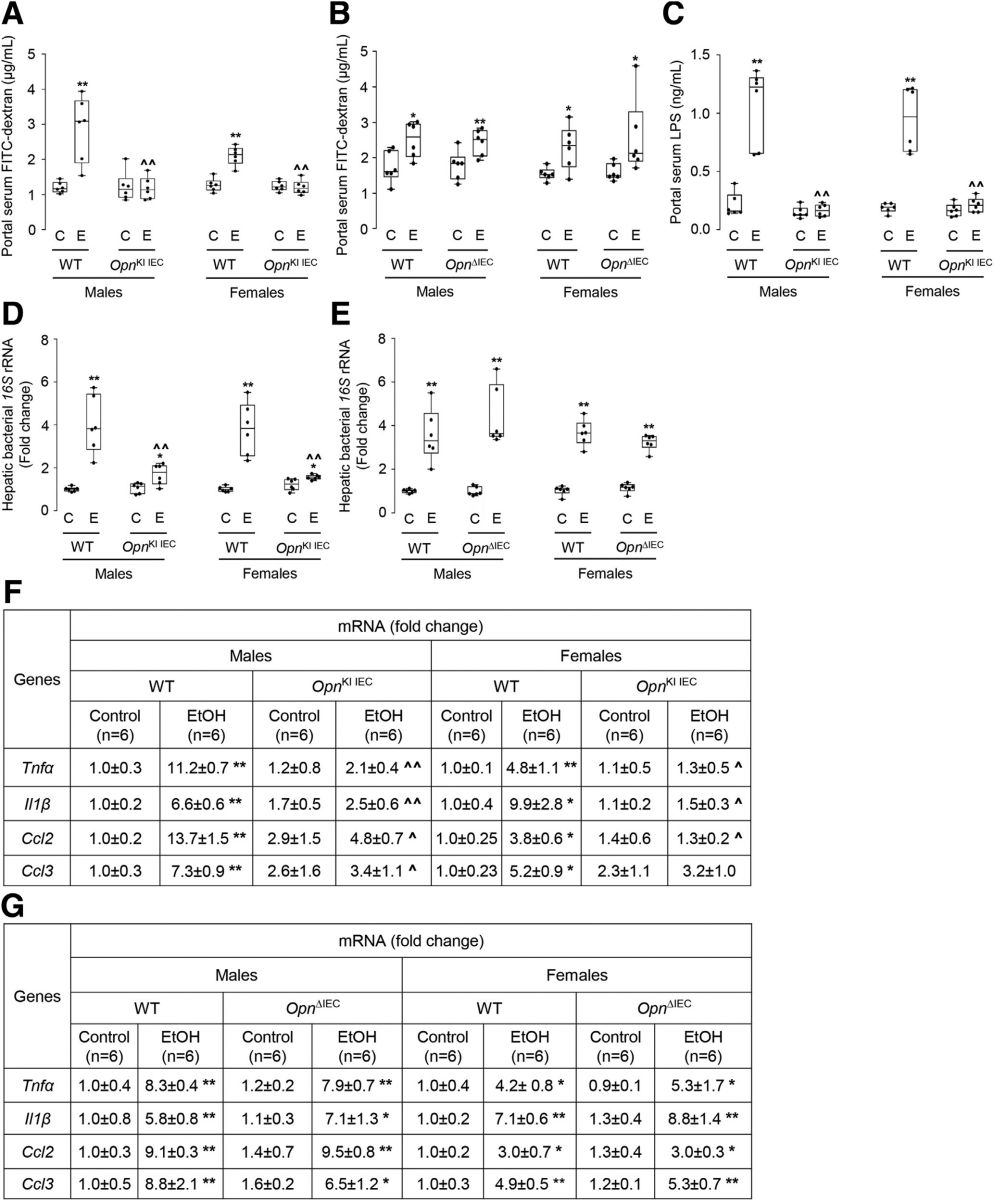
I**ncreased expression of OPN in IECs preserves gut permeability.** WT, *Opn*^KI IEC^ and *Opn*^ΔIEC^ mice were fed control or ethanol diet for 6 weeks to provoke ALD. Portal serum FITC-dextran (*A–B*). Portal serum LPS (*C*). Hepatic bacterial *16S* rRNA. Data were normalized with *18S* as housekeeping gene and FC was calculated against WT control (*D–E*). mRNA expression of pro-inflammatory cytokines in liver. Data were normalized with *β-actin* as housekeeping gene and FC was calculated against WT control (*F–G*). n = 6/group; data are presented as mean ± standard error of the mean. **∗***P* < .05 and **∗∗***P* < .01 vs control; **ˆ***P* < .05 and **ˆˆ***P* < .01 vs WT ethanol. C, Control diet; E, ethanol diet.

Because leaky gut contributes to the pathogenesis of ALD,^3^^,^^18^ we measured gut permeability in these mice. As shown in Figure 2, *A*, portal serum fluorescein isothiocyanate-dextran (FITC-dextran) was lower in ethanol-fed *Opn*^KI IEC^ than in WT mice. Ethanol-fed WT and *Opn*^ΔIEC^ mice showed similar increase in portal serum FITC-dextran compared with their respective control-fed group (Figure 2, *B*). To determine if these changes in gut permeability led to translocation of bacterial products and bacteria from the gut lumen to the portal vein and the liver, we measured the concentration of portal serum LPS and bacterial *16S* rRNA in liver. Ethanol-fed *Opn*^KI IEC^ showed lower LPS and bacterial *16S* rRNA than WT mice (Figure 2, *C–D*). However, hepatic bacterial *16S* rRNA was equally higher in ethanol-fed WT and *Opn*^ΔIEC^ mice compared with their respective control-fed group (Figure 2, *E*). To assess if protection from ALD was due to reduced KC activation, as well as inflammatory cell infiltration and activation, we measured the mRNA expression of pro-inflammatory cytokines in liver. Ethanol-fed *Opn*^KI IEC^ showed lower tumor necrosis factor-α (*Tnfa)*, *Il1b*, *Ccl2*, and *Ccl3* mRNAs than WT mice (Figure 2, *F*) bur remained comparably high in ethanol-fed WT and *Opn*^ΔIEC^ mice (Figure 2, *G*). Overall, these results suggest that overexpression of OPN in IECs preserves the intestinal epithelial barrier function, prevents translocation of LPS, and reduces KC activation and production of pro-inflammatory cytokines, all of which protect from ALD.

### Increased Expression of OPN in IECs Prevents Alcohol-induced Dysbiosis

**Figure 3:**
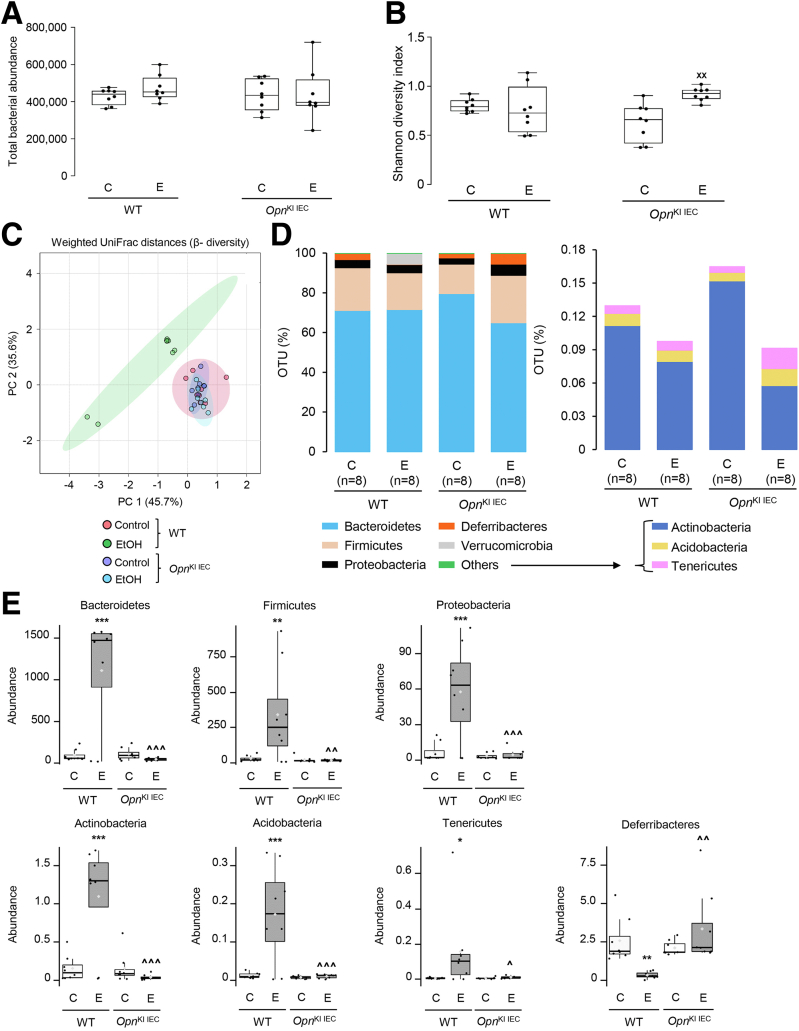

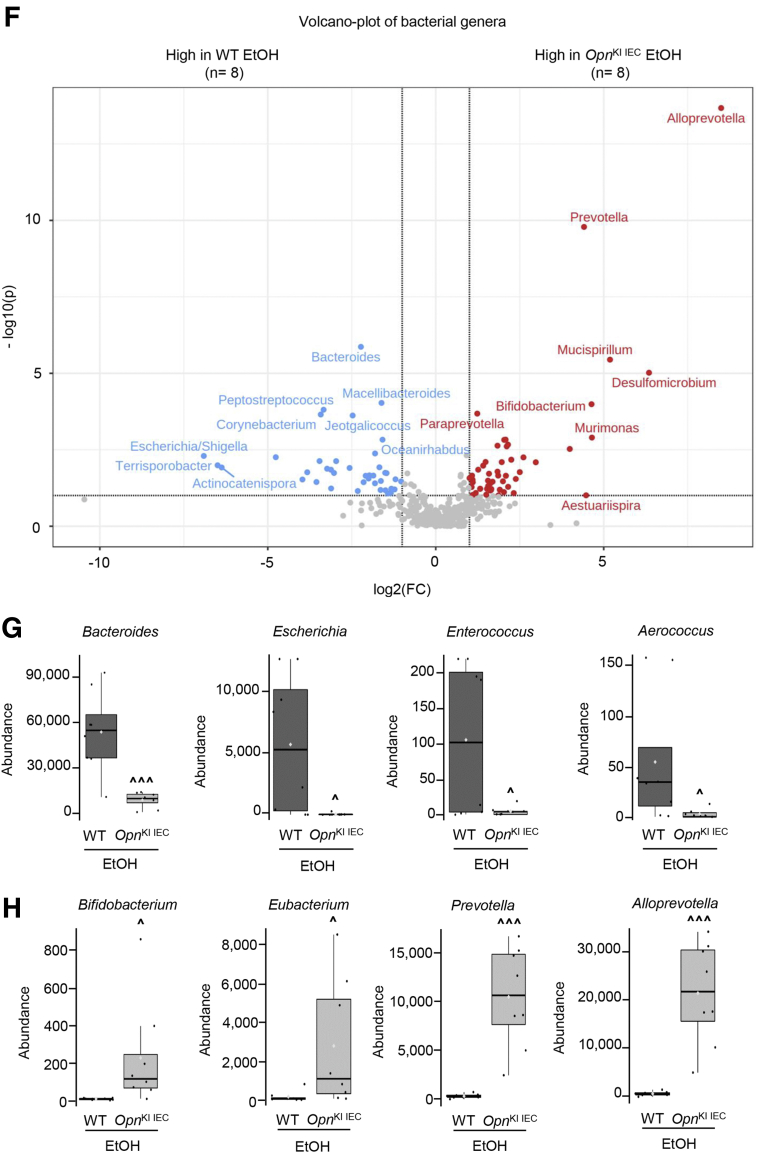
I**ncreased expression of OPN in IECs prevents alcohol-induced dysbiosis.** WT and *Opn*^KI IEC^ mice were fed control or ethanol diet for 6 weeks to provoke ALD; then, 16S rRNA sequencing was performed in stools to analyze the FM. Total bacterial abundance (*A*). Shannon index for alpha diversity (*B*). Principal component analysis plot of weighted UniFrac distances for beta diversity (*C*). Operational taxonomic unit (%) of bacterial phyla (*D*). Differential analysis of bacterial phyla (*E*). Volcano plot of bacterial genera (*F*). Highly abundant bacterial genera in ethanol-fed WT (*G*) and *Opn*^KI IEC^ (*H*) mice. n = 8 (4 males + 4 females)/group. ^**XX**^*P* < .05 vs control; **∗**false discovery rate (FDR) <0.05; **∗∗**FDR <0.01; and **∗∗∗**FDR <0.001 vs control; **ˆ**FDR <0.05; **ˆˆ**FDR <0.01; and **ˆˆˆ**FDR <0.001 vs WT ethanol. C, Control diet; E, ethanol diet.

Alcohol drinking causes intestinal dysbiosis, which disrupts the gut barrier and contributes to ALD.^3^^,^^7^ Thus, we examined if ethanol-fed *Opn*^KI IEC^ mice were protected from intestinal dysbiosis by analyzing the fecal microbiome (FM). Abundance of total bacteria was similar among groups of mice (Figure 3, *A*). The Shannon diversity index (alpha diversity) was higher in ethanol-fed than in control-fed *Opn*^KI IEC^ mice, indicating a diverse and equally distributed microbiome in ethanol-fed *Opn*^KI IEC^ mice (Figure 3, *B*). Weighted UniFrac analysis (beta diversity) revealed that the fecal bacteria in ethanol-fed *Opn*^KI IEC^ was different from WT but was comparable to control-fed *Opn*^KI IEC^ and WT mice (Figure 3, *C*). Eight bacterial phyla were most dominant in all groups of mice (Figure 3, *D*). Abundance of these phyla (Bacteroidetes, Firmicutes, Proteobacteria, Actinobacteria, Acidobacteria, Tenericutes, Deferribacteres) was similar in ethanol-fed and control-fed *Opn*^KI IEC^ but increased in ethanol-fed compared with control-fed WT mice, except for Deferribacteres, which decreased (Figure 3, *E*). Differential analysis of bacterial genera (Figure 3, *F*), showed that 36 genera, including *Bacteroides*, *Escherichia*, *Enterococcus*, and *Aerococcus*, all pathogenic and increased in ALD, were higher in ethanol-fed WT than in *Opn*^KI IEC^ mice (Figure 3, *G*). Twenty-two genera, including *Bifidobacterium*, *Eubacterium*, *Prevotella*, and *Alloprevotella*, linked to intestinal Trp and SCFAs metabolism and known to be beneficial, were higher in ethanol-fed *Opn*^KI IEC^ than in WT mice (Figure 3, *H*). Overall, these findings suggest that *Opn*^KI IEC^ mice are protected from alcohol-induced intestinal dysbiosis.

### Intestinal Microbiome (IM) From *Opn*^KI IEC^ Mice Protects From ALD

**Figure 4:**
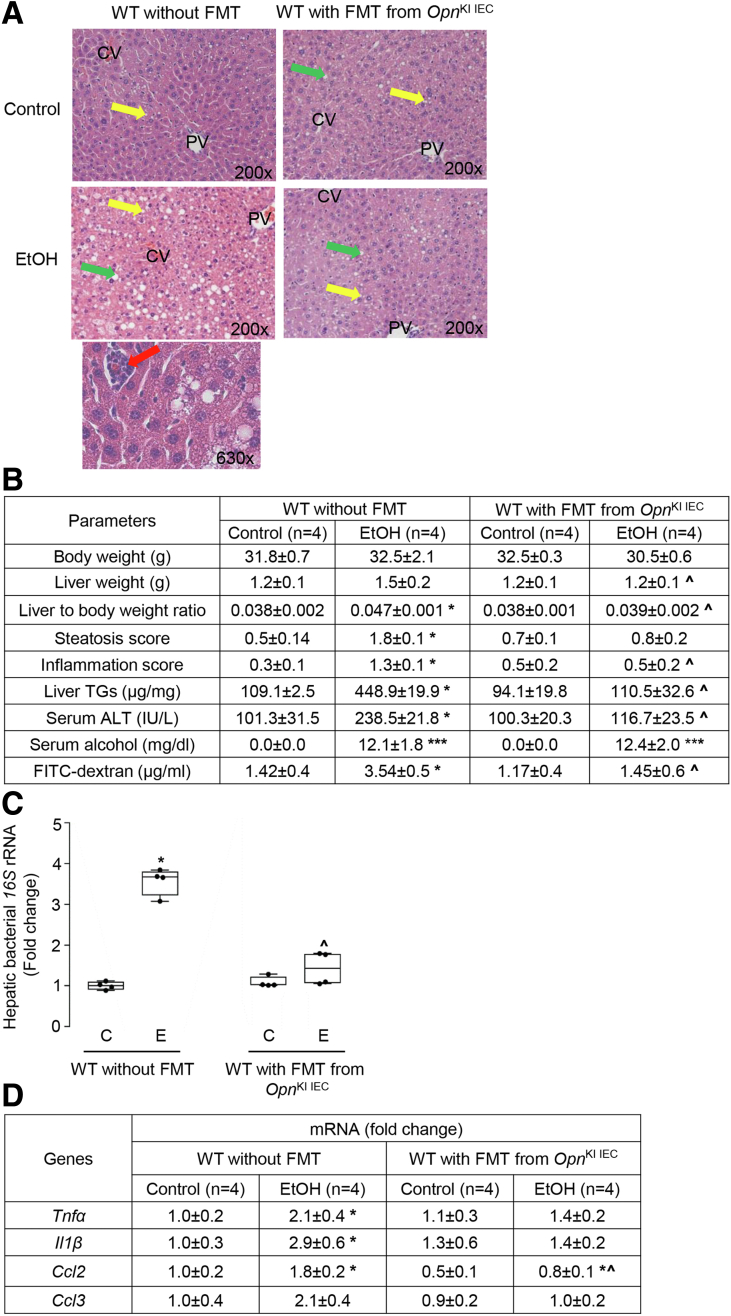
**IM from *Opn***^**KI IEC**^**mice protects from ALD.** WT mice were transplanted with FM from *Opn*^KI IEC^ and fed control or ethanol diet for 6 weeks to provoke ALD. Liver H&E staining (*green arrows*, macrovesicular steatosis; *yellow arrows*, microvesicular steatosis; *red arrows*, inflammatory foci; CV, central vein; PV, portal vein) (*A*). Body weight, liver weight, liver to body weight ratio, pathology scores, liver TGs, serum ALT activity, serum alcohol levels, and portal serum FITC-dextran (*B*). Hepatic bacterial *16S* rRNA. Data were normalized with *18S* as housekeeping gene and FC was calculated against no-FMT control (*C*). mRNA expression of hepatic pro-inflammatory cytokines. Data were normalized with *β-actin* as housekeeping gene and FC was calculated against no-FMT control (*D*). n = 4/group; data are expressed as mean ± standard error of the mean. **∗***P* < .05 and **∗∗∗***P* < .001 vs control; **ˆ***P* < .05 vs no-FMT ethanol. C, Control diet; E, ethanol diet.

To determine if IM from *Opn*^KI IEC^ protects from ALD, we transplanted FM from *Opn*^KI IEC^ to WT mice and fed them with control or ethanol diet for 6 weeks. H&E staining (Figure 4, *A*), the steatosis and inflammation scores, liver TGs, and serum ALT activity (Figure 4, *B*), showed less alcohol-induced liver injury in WT after fecal microbiome transplant (FMT) from *Opn*^KI IEC^ mice compared with mice without FMT. The increase in portal serum FITC-dextran in ethanol-fed WT mice was prevented by FMT from *Opn*^KI IEC^ mice (Figure 4, *B*). Ethanol-fed WT with FMT from *Opn*^KI IEC^ mice showed lower bacterial *16S* rRNA (Figure 4, *C*), and decreased mRNA expression of pro-inflammatory cytokines (*Tnfa*, *Il1b*, *Ccl2*, *Ccl3*) in liver compared with mice without FMT (Figure 4, *D*). The duodenum, jejunum, ileum, and colon from these mice did not show structural changes after FMT and ethanol feeding (not shown). These data indicate that IM from *Opn*^KI IEC^ mice improves intestinal epithelial barrier function, lowers hepatic pro-inflammatory cytokines, and protects from ALD.

### Overexpression of OPN in IECs Preserves the IM and Protects From ALD

**Figure 5:**
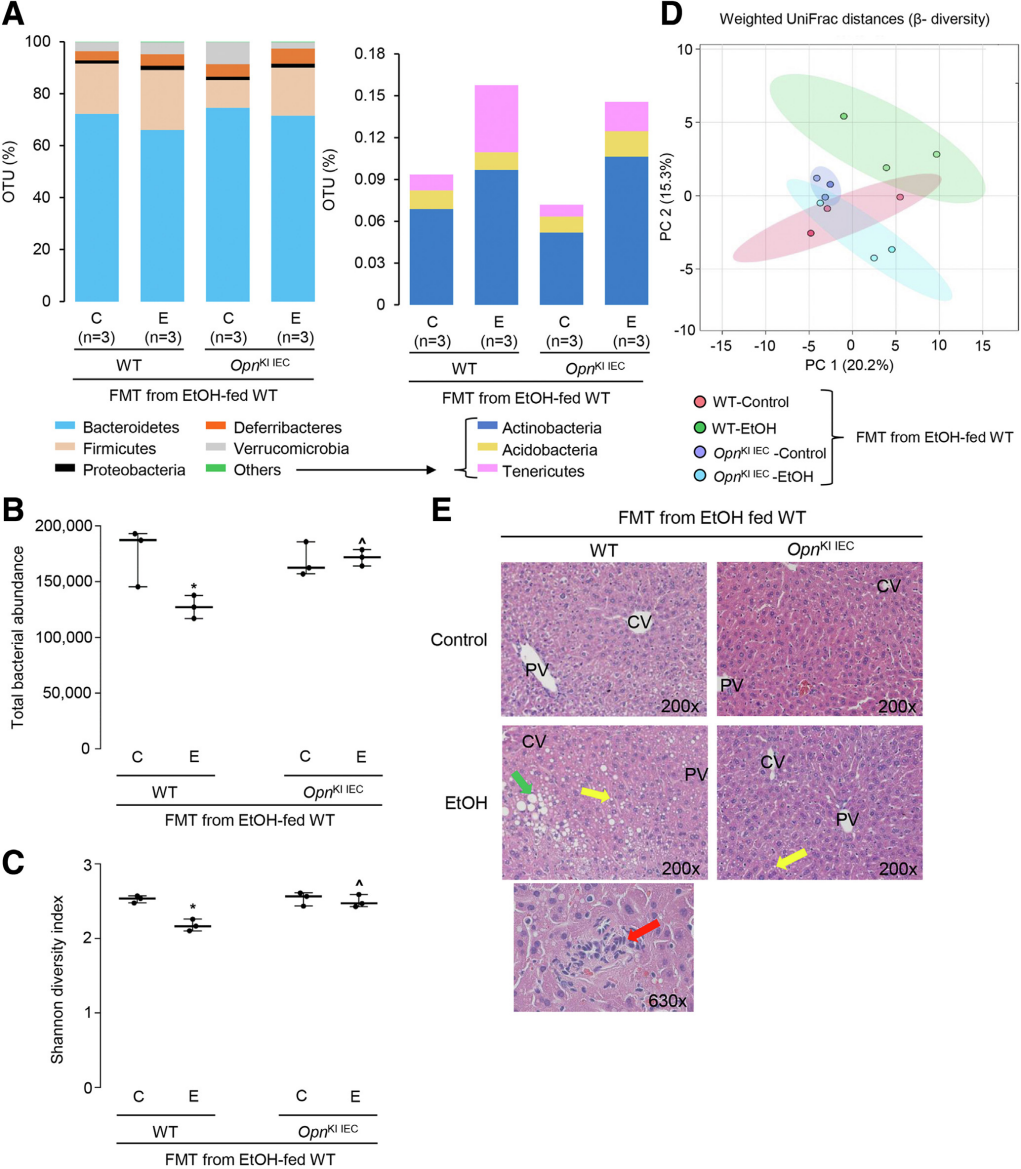

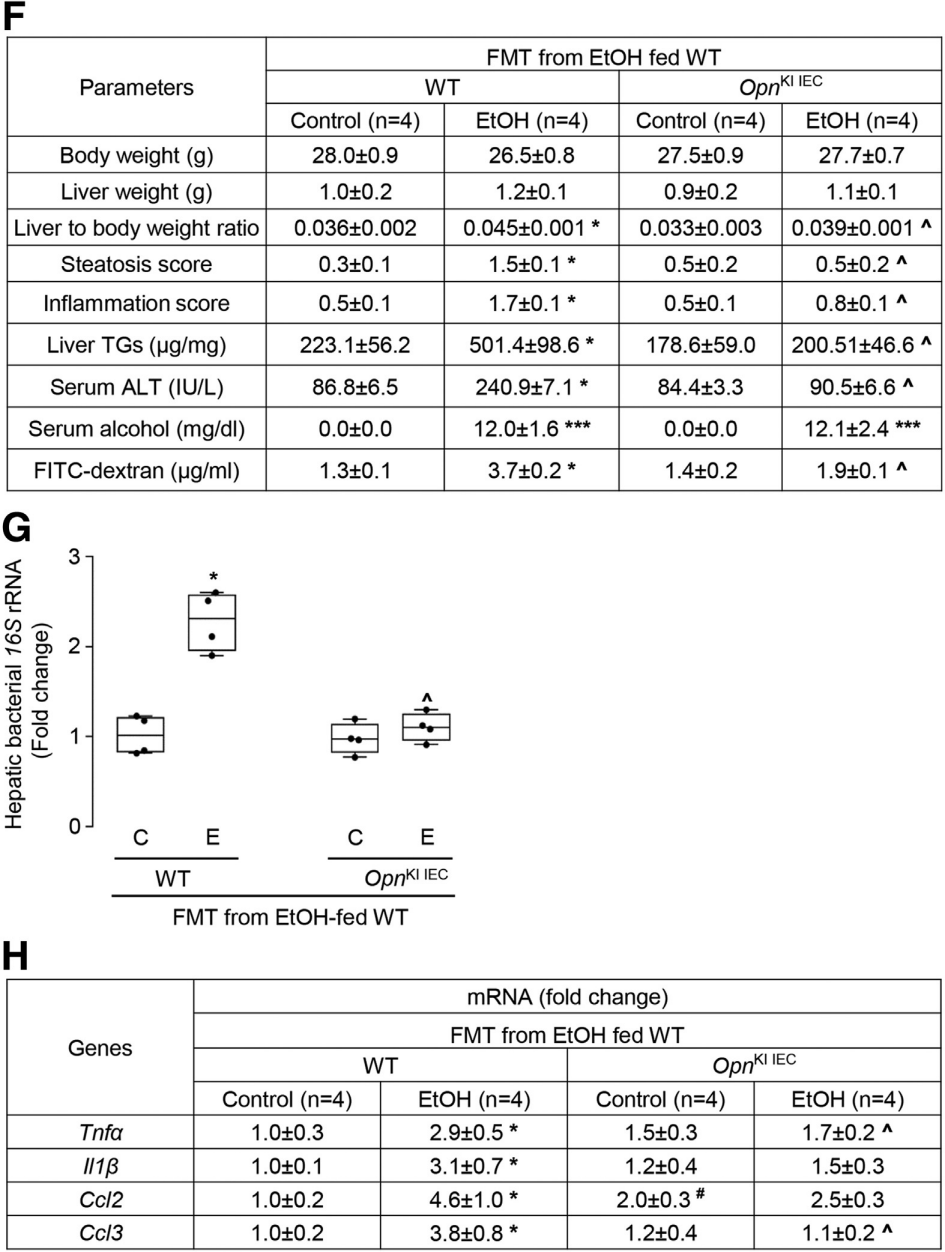
**Overexpression of OPN in IECs preserves the gut microbiome and protects from ALD.** FM from 6-week ethanol-fed WT were transplanted to WT and *Opn*^KI IEC^ mice, and then mice were fed control or ethanol diet for 6 weeks to provoke ALD. 16S rRNA sequencing was performed to analyze the FM. Operational taxonomic unit (%) of bacterial phyla (*A*). Total bacterial abundance (*B*). Shannon Index for alpha diversity (*C*). Principal component analysis plot of weighted UniFrac distances for beta diversity (*D*). Liver H&E staining (*green arrows*, macrovesicular steatosis; *yellow arrows*, microvesicular steatosis; *red arrows*, inflammatory foci; CV, central vein; PV, portal vein) (*E*). Body weight, liver weight, liver to body weight ratio, pathology scores, liver TGs, serum ALT activity, serum alcohol levels, and portal serum FITC-dextran (*F*). Hepatic bacterial *16S* rRNA. Data were normalized with *18S* as housekeeping gene and FC was calculated against WT control (*G*). Hepatic mRNA expression of pro-inflammatory cytokines. Data were normalized with *β-actin* as housekeeping gene, and FC was calculated against WT control (*H*). n = 3–4/group; data are expressed as mean ± standard error of the mean. **∗***P* < .05 and **∗∗∗***P* < .001 vs control; ^**#**^*P* < .05 vs WT control; **ˆ***P* < .05 vs WT ethanol. C, Control diet; E, ethanol diet.

To examine if overexpression of OPN in IECs changes the IM in *Opn*^KI IEC^ mice, we performed FMT from ethanol-fed WT mice to *Opn*^KI IEC^ mice and fed them control or ethanol diet for 6 weeks to provoke ALD. FM analysis showed that 8 bacterial phyla were the most prevalent in these mice (Figure 5, *A*). Ethanol-fed *Opn*^KI IEC^ with FMT from ethanol-fed WT mice showed higher total bacteria and alpha diversity compared with ethanol-fed WT with FMT from ethanol-fed WT mice (Figure 5, *B–C*). Beta diversity analysis showed that the IM in ethanol-fed *Opn*^KI IEC^ with FMT from ethanol-fed WT mice was different from that of ethanol-fed WT with FMT from ethanol-fed WT mice (Figure 5, *D*). These results suggest that overexpression of OPN in IECs regulates the IM. To assess if the OPN-mediated regulation of the IM protects from ALD, we evaluated liver injury. H&E staining (Figure 5, *E*), the steatosis and inflammation scores, liver TGs, and serum ALT activity (Figure 5, *F*) demonstrated less liver injury in ethanol-fed *Opn*^KI IEC^ with FMT from ethanol-fed WT mice compared with ethanol-fed WT with FMT from ethanol-fed WT mice. The former showed decreased portal serum FITC-dextran, less bacterial *16S* rRNA in liver, and lower hepatic mRNA expression of *Tnfa*, *Il1b*, *Ccl2*, and *Ccl3*, compared with the latter (Figure 5, *F–H*), indicating improved gut barrier function in *Opn*^KI IEC^ mice. The duodenum, jejunum, ileum, and colon from these mice did not show structural changes (not shown). Thus, these results indicate that overexpression of OPN in IECs preserves the gut microbiome, the intestinal epithelial barrier function, and protects from ALD.

### OPN Preserves the Gut Microbiome by Inducing AMPs Expression in IECs, Which Maintains Intestinal Barrier Function and Protects From ALD

**Figure 6:**
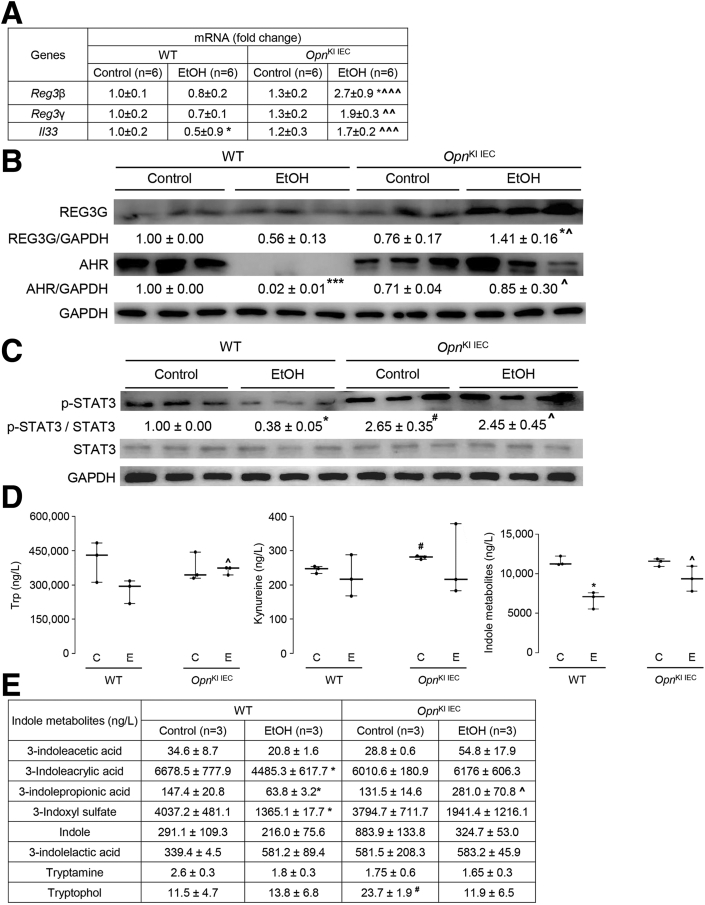

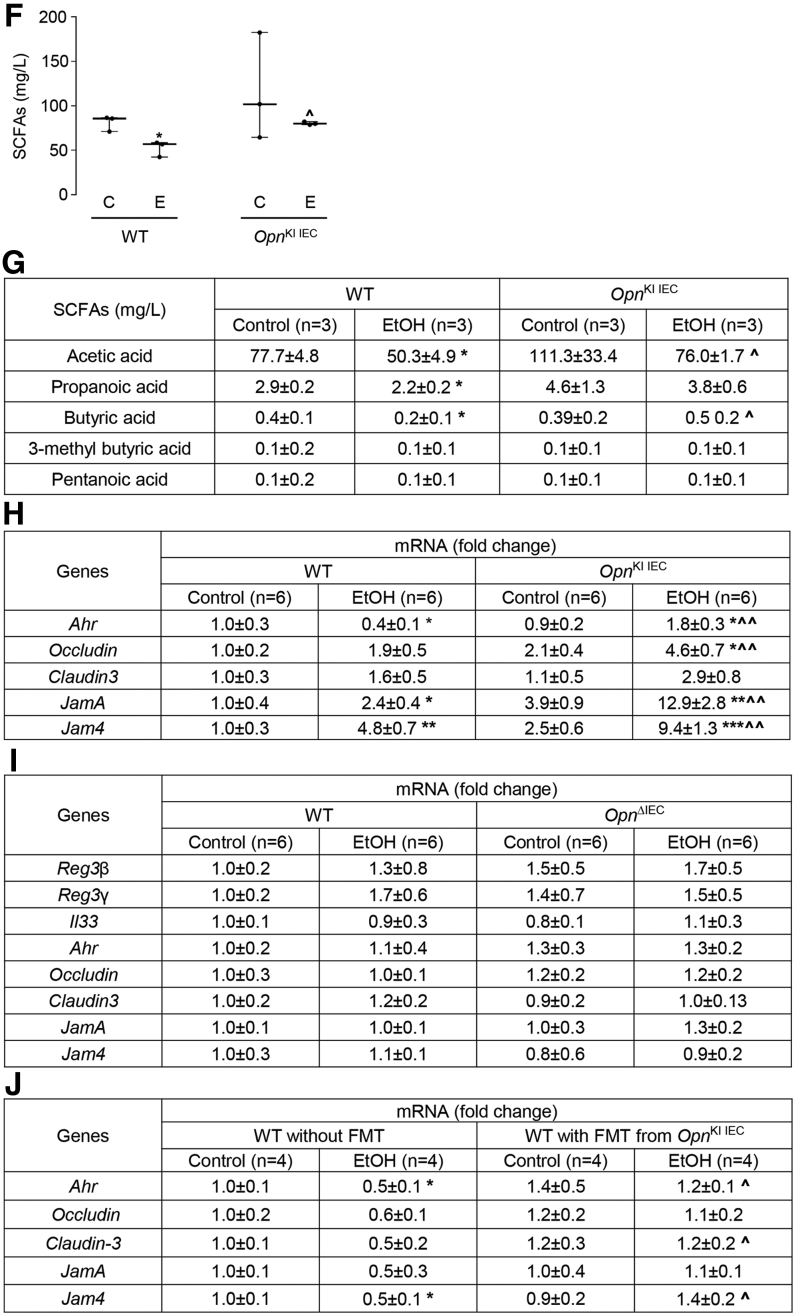

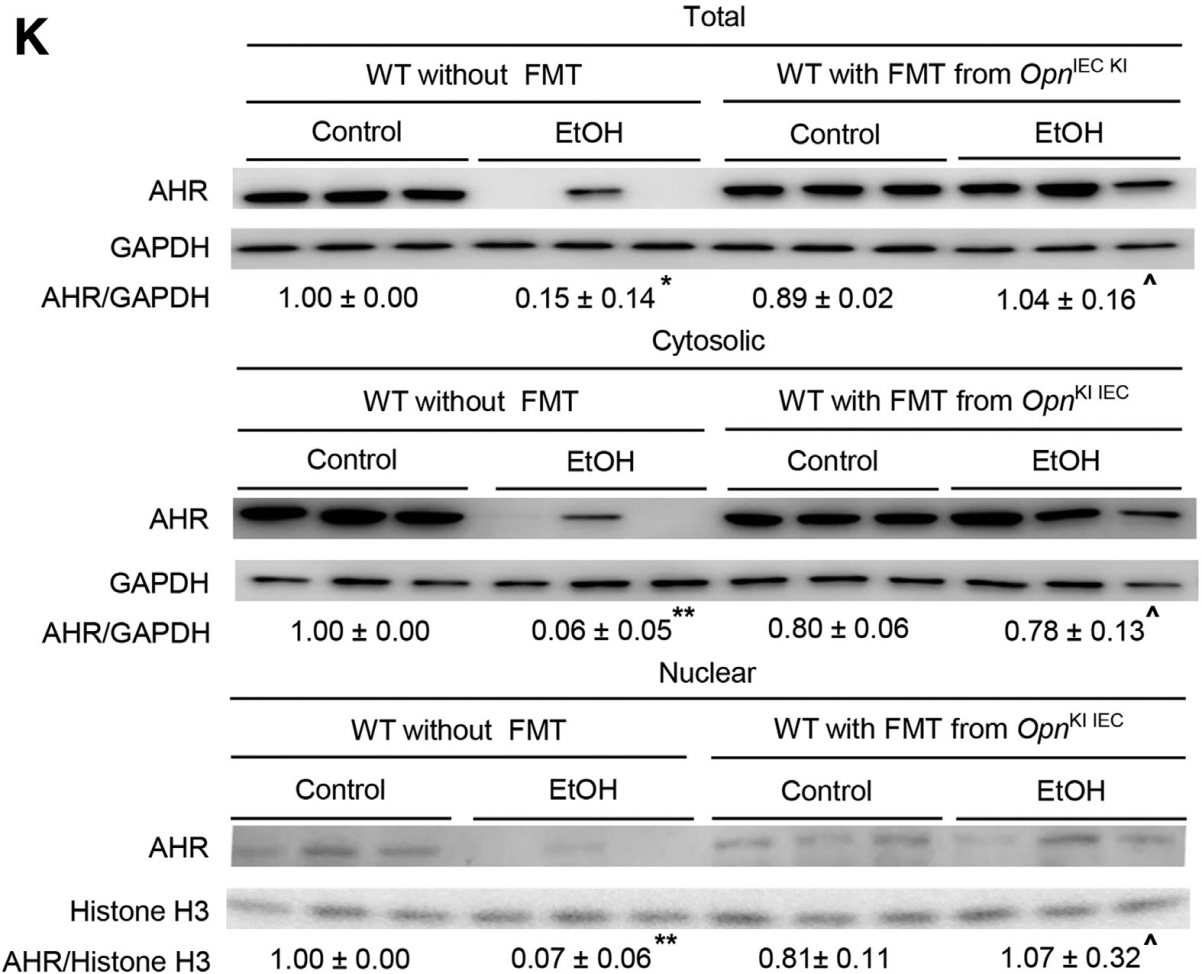
**OPN preserves the gut microbiome by inducing AMPs expression in IECs, which maintainsintestinal barrier function and protects from ALD.** WT and *Opn*^KI IEC^ mice were fed control or ethanol diet for 6 weeks to provoke ALD. mRNA expression of *Reg3β*, *Reg3γ*, and *Il33* in IECs isolated from jejunum. Data were normalized with *Gapdh* as housekeeping gene and FC was calculated against WT control (n = 6 [3 males + 3 females]/group) (*A*). Western blot of REG3G and total AHR in IECs isolated from jejunum (n = 3 males/group) (*B*). Western blot of STAT3 and pSTAT3 in IECs isolated from jejunum (n = 3 males/group) (*C*). Levels of Trp, its metabolites kynureine and indole metabolites (3-indoleacetic acid + 3-indoleacrylic acid + 3-indolepropionic acid + 3-indoxyl sulfate + indole + indole-3-lactic acid + tryptamine + tryptophol) in portal serum (n = 3 males/group) (*D–E*). Levels of SCFAs (acetic acid + propionic acid + butyric acid) in portal serum (n = 3 males/group) (*F–G*). mRNA expression of *Ahr*, *Occludin*, *Claudin3*, *JamA*, and *Jam4* in IECs from jejunum. Data were normalized with *Gapdh* as housekeeping gene and FC was calculated against WT control (n = 6 [3 males +3 females]/group) (*H*). Data are expressed as mean ± standard error of the mean (SEM). **∗***P* < .05; **∗∗***P* < .01; and **∗∗∗***P* < .001 vs control; ^**#**^*P* < .01 vs WT control; **ˆ***P* < .05; **ˆˆ***P* < .01; and **ˆˆˆ***P* < .01 vs WT ethanol. WT and *Opn*^ΔIEC^ mice were fed control or ethanol diet for 6 weeks to provoke ALD. mRNA expression of *Reg3β*, *Reg3γ*, *Il33*, *Ahr*, *Occludin*, *Claudin3*, *JamA*, and *Jam4* in IECs from jejunum. Data were normalized with *Gapdh* as housekeeping gene, and FC was calculated against WT control. n = 6 (3 males + 3 females)/group; data are expressed as mean ± SEM (*I*). WT mice were transplanted with FM from *Opn*^KI IEC^ and were fed the control or ethanol diet for 6 weeks to provoke ALD. mRNA expression of *Ahr*, *Occludin*, *Claudin3*, *JamA*, and *Jam4* in IECs from jejunum. Data were normalized with *Gapdh* as housekeeping gene, and FC was calculated against no-FMT control (n = 4/group) (*J*). Western blot of total, cytosolic, and nuclear Ahr in IECs from jejunum (n = 3/group) (*K*). Data are expressed as mean ± SEM. **∗***P* < .05 and **∗∗***P* < .01 vs control; **ˆ***P* < .05 vs no-FMT ethanol. C, Control diet; E, ethanol diet.

To understand how IEC-derived OPN preserves the gut microbiome upon alcohol exposure, we examined AMPs expression in IECs from jejunum. Regenerating islet-derived protein *(Reg) 3β* and *3γ* mRNA and protein were higher in IECs from ethanol-fed *Opn*^KI IEC^ than in WT mice (Figure 6, *A–B*). Interleukin-33 (*Il33)* and phosphorylation of STAT3, known to enhance AMPs in IECs,^19^ were higher in jejunal IECs from control and ethanol-fed *Opn*^KI IEC^ mice, whereas *Il33* was higher only in jejunal IECs from ethanol-fed *Opn*^KI IEC^ mice (Figure 6, *A and C*). To determine if the effect of OPN in the gut microbiome preserved the intestinal barrier function, first we measured portal serum Trp metabolites and SCFAs, produced by the gut microbiome, and known to improve gut barrier function.^8^^,^^20^ Second, we evaluated the mRNA expression of aryl hydrocarbon receptor (*Ahr*), which regulates gut barrier function via Trp metabolites^8^ and SCFAs.^21^ Third, we analyzed TJs mRNA expression in IECs from jejunum. Trp, indole metabolites, and SCFAs increased in portal serum from ethanol-fed *Opn*^KI IEC^ compared with WT mice (Figure 6, *D–G*). Ethanol-fed *Opn*^KI IEC^ showed higher *Ahr*, *Occludin*, junctional adhesion molecule A (*JamA*), and junctional adhesion molecule 4 (*Jam4*) mRNA expression in IECs from jejunum, than in WT mice (Figure 6, *H*). AHR protein expression was similar in IECs from jejunum in control and ethanol-fed *Opn*^KI IEC^ mice (Figure 6, *B*).

Next, to establish the link among OPN, Ahr, and TJ proteins, the mRNA expression of AMPs (*Reg3β* and *Reg3γ*), *Il33*, *Ahr*, and TJ proteins (*Occludin*, *Claudin3*, *JamA*, *Jam4*) were analyzed in IECs from jejunum of *Opn*^ΔIEC^ and WT mice fed control or ethanol diet for 6 weeks. *Opn*^ΔIEC^ mice did not show meaningful changes in *Reg3β*, *Reg3γ*, *Il33*, *Ahr*, *Occludin*, *Claudin3*, *JamA*, and *Jam4* after ethanol feeding (Figure 6, *I*). To confirm that the FM from *Opn*^KI IEC^ mice preserved the intestinal barrier function, the expression of *Ahr*, *Occludin*, *Claudin3*, *JamA*, and *Jam4* was measured in IECs from jejunum of WT with FMT from *Opn*^KI IEC^ mice. Ethanol-fed WT with FMT from *Opn*^KI IEC^ mice showed higher mRNA expression of *Ahr*, *Occludin*, *Claudin3*, *JamA*, and *Jam4* compared with ethanol-fed WT mice with no FMT (Figure 6, *J*). Analysis of total, cytosolic, and nuclear protein revealed that ethanol lowered AHR expression in IECs from jejunum and that FMT from *Opn*^KI IEC^ mice preserved protein expression (Figure 6, *K*). These data indicate that overexpression of OPN in IECs increases intestinal AMPs to maintain the gut microbiome, which maintains intestinal barrier function via Trp metabolites, SCFAs, and Ahr, all of which protect *Opn*^KI IEC^ mice from ALD.

### Oral Administration of mOPN maintains the gut microbiome and protects from ALD

**Figure 7:**
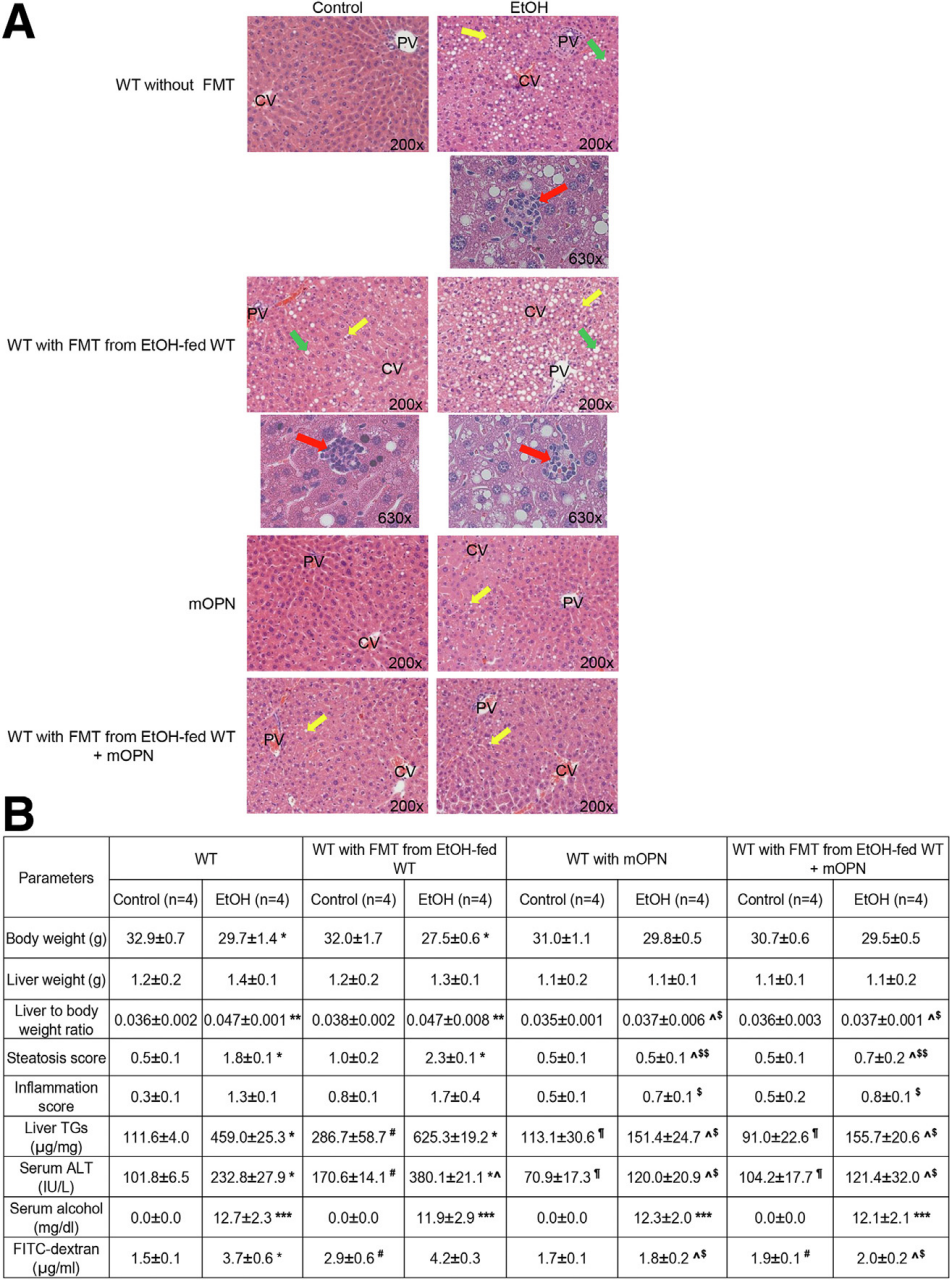

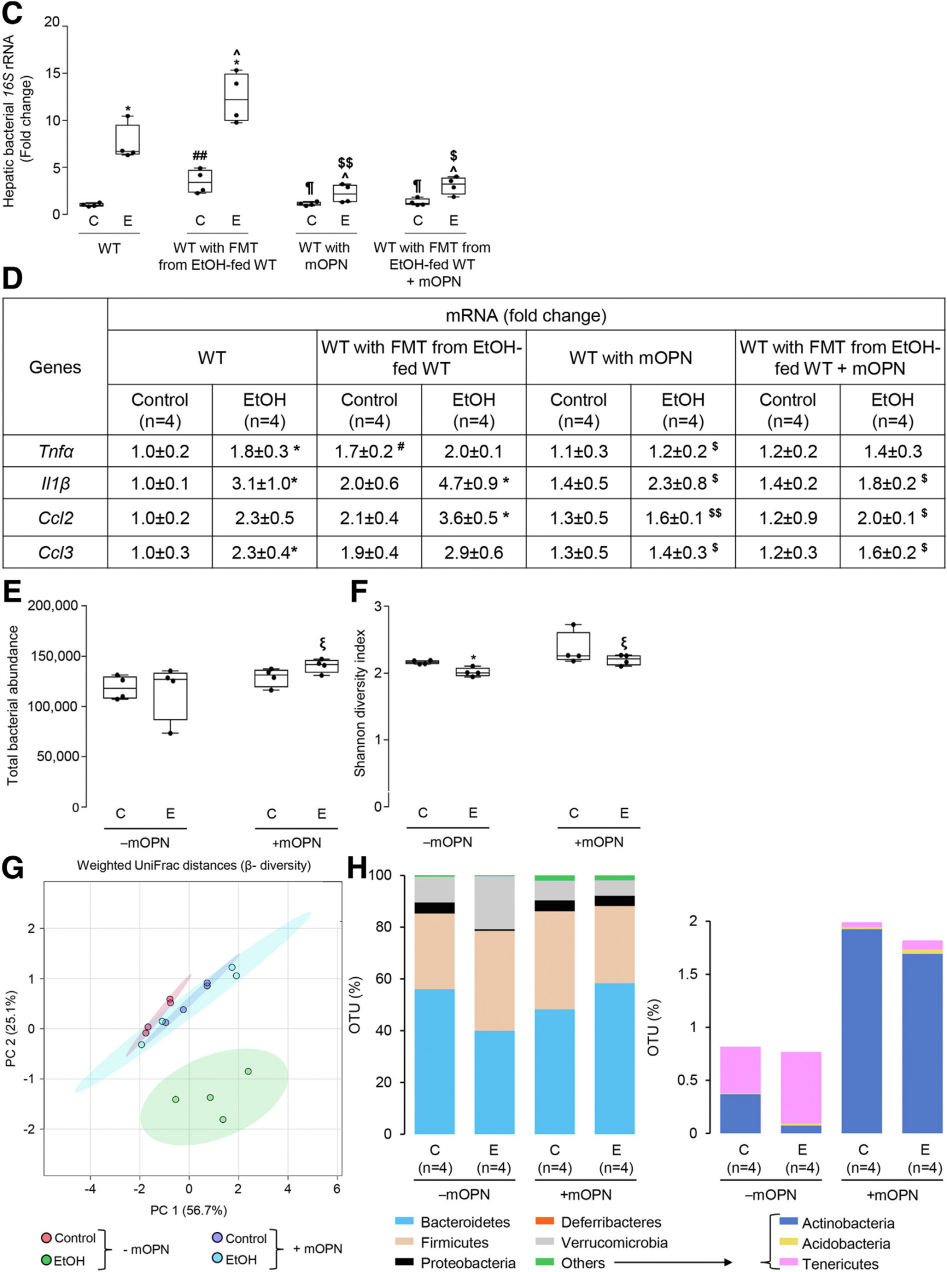

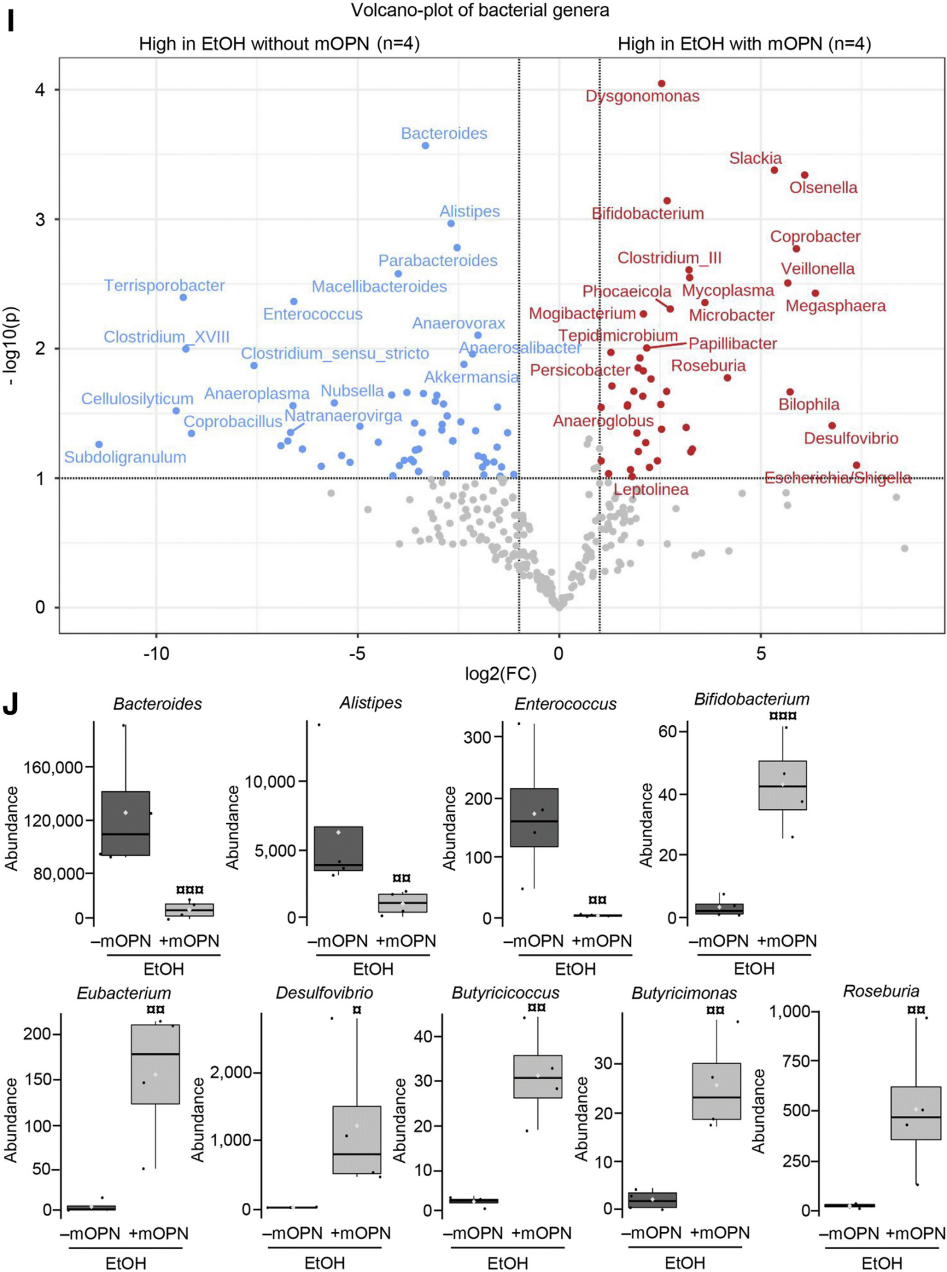
**Oral administration of mOPN preserves the gut microbiome and protects from ALD.** FM from 6-week ethanol-fed WT mice was transplanted to WT mice, and then mice were fed control or ethanol diet for 6 weeks in the presence or absence of mOPN. Liver H&E staining (*green arrows*, macrovesicular steatosis; *yellow arrows*, microvesicular steatosis; *red arrows*, inflammatory foci; CV, central vein; PV, portal vein) (*A*). Body weight, liver weight, liver to body weight ratio, pathology scores, liver TGs, serum ALT activity, serum alcohol levels, and portal serum FITC-dextran (*B*). Hepatic bacterial *16S* rRNA. Data were normalized with *18S* as housekeeping gene, and FC was calculated against WT control (*I*). Liver mRNA expression of pro-inflammatory cytokines. Data were normalized with *β-actin* as housekeeping gene, and FC was calculated against WT control (*D*). Total bacterial abundance (*E*). Shannon Index for alpha diversity (*F*). Principal component analysis plot of weighted UniFrac distances for beta diversity (*G*). Operational taxonomic unit (%) of bacterial phyla (*H*). Volcano plot of bacterial genera (*I*). Differential analysis of bacterial genera (*J*). n = 4/group; data are presented as mean ± standard error of the mean. **∗***P* < .05; **∗∗***P* < .01; and **∗∗∗***P* < .001 vs control; ^**#**^*P* < .05 vs no-FMT control; ^**¶**^*P* < .05 vs FMT control; **ˆ***P* < .05 vs no-FMT ethanol; ^**$**^*P* < .05 and ^**$$**^*P* < .01 vs FMT ethanol. ^**ξ**^*P* < .05 vs without mOPN ethanol. ^**¤**^False discovery rate (FDR) <0.05; ^**¤¤**^FDR <0.01; and ^**¤¤¤**^FDR <0.001 vs without mOPN ethanol. C, Control diet; E, ethanol diet.

Our previous report proved that oral administration of mOPN during chronic ethanol feeding ameliorates ALD.^1^ To dissect if this occurred by preserving the gut microbiome, we performed FMT from ethanol-fed WT to WT mice, and fed them with control or ethanol diet for 6 weeks in the presence or absence of mOPN. H&E staining (Figure 7, *A*), the steatosis and inflammation scores, liver TGs, serum ALT activity, portal serum FITC-dextran (Figure 7, *B*), hepatic bacterial *16S* rRNA (Figure 7, *C*), and hepatic mRNA expression of pro-inflammatory cytokines (Figure 7, *D*) revealed less liver injury in ethanol-fed WT with oral administration of mOPN in the presence or absence of FMT from ethanol-fed WT mice. The duodenum, jejunum, ileum, and colon from these mice did not show structural changes (not shown). To understand if protection from ALD by mOPN was mediated by preserving the gut microbiome, we analyzed the FM from WT mice fed control or ethanol diet in the presence or absence of mOPN. Abundance of total bacteria and alpha diversity was higher in ethanol-fed WT mice with mOPN than in mice without mOPN (Figure 7, *E–F*), suggesting that mOPN maintains a rich, diverse, and equally distributed microbiome. Analysis of beta diversity showed that fecal bacteria in mOPN plus ethanol-fed WT was different than that without mOPN but was comparable to control-fed WT with or without mOPN (Figure 7, *G*). Eight bacterial phyla were most abundant in mice with or without mOPN (Figure 7, *H*). Differential analysis of bacterial genera (Figure 7, *I*) showed that chronic ethanol feeding without mOPN promoted growth of pathogenic genera, such as *Bacteroides*, *Alistipes*, and *Enterococcus* (Figure 7, *J*). However, mOPN promoted growth of bacteria, such as *Bifidobacterium*, *Eubacterium*, *Desulfovibrio*, *Butyricicoccus*, *Butyricimonas*, and *Roseburia*, linked to intestinal Trp and SCFAs metabolism (Figure 7, *J*), which play a beneficial role in the gut.^8^^,^^22^, ^23^, ^24^ These findings suggest that mOPN protects from ALD by preserving the gut microbiome.

### Oral Administration of mOPN Induces AMPs in IECs, Which Preserve the Gut Microbiota and the Intestinal Barrier Function

**Figure 8:**
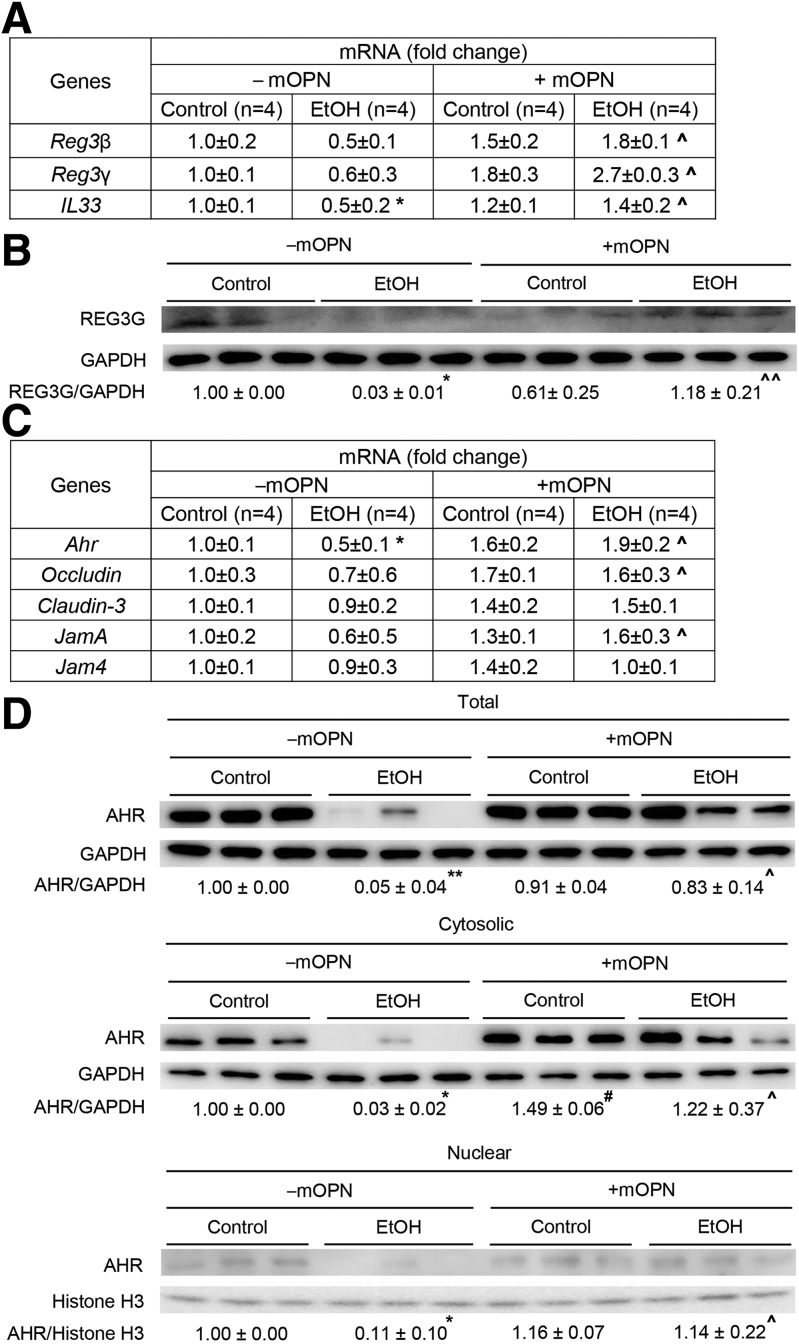

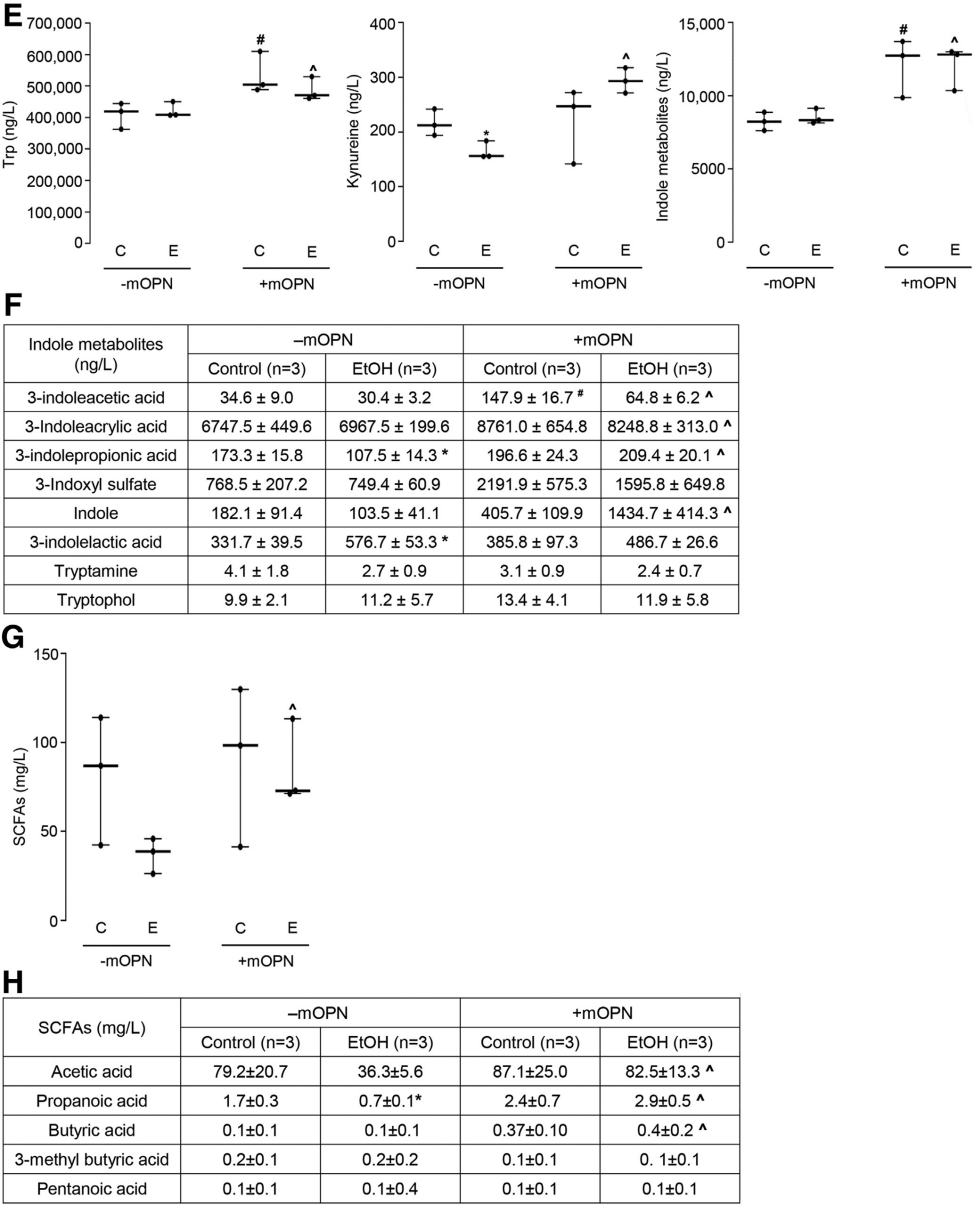
**Oral administration of mOPN induces AMPs in IECs, which preserve the gut microbiota and the intestinal barrier function.** WT mice were fed control or ethanol diet for 6 weeks in the presence or absence of mOPN. mRNA expression of *Reg3β*, *Reg3γ*, and *Il33* in IECs from jejunum. Data were normalized with *Gapdh* as housekeeping gene, and FC was calculated against without mOPN control (n = 4/group) (*A*). REG3G protein expression in IECs from jejunum (n = 3/group) (*B*). mRNA expression of *Ahr*, *Occludin*, *Claudin3*, *JamA*, and *Jam4* in IECs from jejunum. Data were normalized with *Gapdh* as housekeeping gene, and FC was calculated against without mOPN control (n = 4/group) (*C*). Western blot of total, cytosolic and nuclear AHR in IECs from jejunum (n = 3/group) (*D*). Levels of Trp, its metabolites kynureine and indole metabolites (3-indoleacrylic acid + 3-indoleacrylic acid + 3-indolepropionic acid + 3-indoxyl sulfate + indole + indole-3-lactic acid + tryptamine + tryptophol) in portal serum (n = 3/group) (*E–F*). Level of SCFAs (acetic acid + propionic acid + butyric acid) in portal serum (n = 4/group) (*G–H*). Data are expressed as mean ± standard error of the mean. **∗***P* <0 .05 and **∗∗***P* < .01 vs control; ^**#**^*P* < .05 vs without mOPN control; **ˆ***P* < .05 vs without mOPN ethanol. C, Control diet; E, ethanol diet.

To understand if mOPN preserves the gut microbiome by inducing AMPs in IECs, we analyzed the expression of *Reg 3β*, *Reg 3γ*, and *Il33* in IECs from jejunum. Chronic ethanol feeding lowered *Reg3β*, *Reg3γ*, and *Il33* mRNAs*,* and mOPN induced them in IECs from jejunum (Figure 8, *A*). Similar findings occurred for REG3G protein (Figure 8, *B*). Moreover, mOPN increased mRNA expression of *Ahr* and TJ proteins (*Occludin*, *Claudin3*, *JamA*) in IECs from jejunum (Figure 8, *C*). Analysis of total, cytosolic, and nuclear AHR protein in IECs from jejunum revealed that mOPN prevented the ethanol-mediated decrease in AHR (Figure 8, *D*). WT mice fed ethanol and mOPN also showed higher levels of Trp, its metabolites, and SCFAs in portal serum (Figure 8, *E–H*). In summary, these findings indicate that both overexpression of OPN in IECs and oral administration of mOPN during chronic ethanol feeding preserve the gut microbiome, intestinal barrier integrity, and protect from ALD through similar mechanisms.

## Discussion

The main findings of this study are that overexpression of OPN in the IECs protects from ALD and that oral administration of mOPN has therapeutic potential to prevent or delay ALD. In both cases, protection from ALD occurs by preserving the gut microbiome and the intestinal epithelial barrier function.

A key finding is that OPN prevents alcohol-induced bacterial dysbiosis in the intestine, central to the pathogenesis of ALD.^3^^,^^25^^,^^26^ Intestinal dysbiosis participates in the onset and progression of ALD as a result of effects in the gut-liver axis.^3^^,^^25^ The intestinal epithelium is in close contact with pathogenic and beneficial microorganisms.^27^^,^^28^ The host evolves unique regulatory mechanisms to maintain a homeostatic balance between tolerance and immunity to the microbiota.^19^ A vital regulatory mechanism is intestinal AMPs, such as Reg3β and Reg3γ, mainly produced by IECs and Paneth cells.^19^^,^^29^ Alcohol drinking downregulates intestinal AMPs, causing quantitative and qualitative changes in the IM that lead to leaky gut and promote ALD.^5^ In this study, we show that overexpressing OPN in IECs or administering oral mOPN prevent the ethanol-mediated reduction in AMPs in IECs and therefore protect from ALD. To understand how OPN induces the expression of AMPs in IECs, we evaluated the mRNA expression of G protein-coupled receptor 43 (*Gpr43*),^30^ stimulator of interferon genes (*Sting*),^31^ and *Il33*,^19^ known to promote antimicrobial peptide expression in IECs. *Gpr43* and *Sting* did not change in IECs from jejunum of *Opn*^KI IEC^ (not shown). Interestingly, ethanol-fed *Opn*^KI IEC^ mice and mOPN plus ethanol-fed WT mice had increased *Il33* expression in jejunal IECs. Moreover *Opn*^KI IEC^ mice showed high level of pSTAT3 in jejunal IECs. Studies have demonstrated that pSTAT3, under IL33 induction or alone, induce *Reg3β* and *Reg3γ* in IECs.^19^^,^^30^ These results suggest that OPN may induce AMPs expression in IECs by pSTAT3 through IL33 signaling, or directly induces STAT3 phosphorylation in IECs. However, the specific effect of OPN on IL33 or STAT3 in IECs to induce production of AMPs needs further investigation. More importantly, this study reveals that the IM in *Opn*^KI IEC^ mice is resistant to alcohol-induced bacterial dysbiosis and explains why they are protected from ALD. This is also supported by less alcohol-induced liver injury in *Opn*^KI IEC^ and WT with FMT from *Opn*^KI IEC^ mice.

A second finding is that OPN preserves the gut microbiome and the intestinal epithelial barrier function by maintaining TJs, and therefore, protects from ALD. The intestinal epithelium, a physical barrier of IECs sealed through TJs,^27^^,^^32^ maintains gut barrier integrity and is the first line of defense against bacterial toxins and pathogens in the intestinal lumen.^32^^,^^33^ Intestinal bacteria are involved in metabolism^8^^,^^34^ and signal to IECs to maintain gut barrier integrity.^8^ An essential player in this scenario is the Ahr, which supports TJs in IECs^8^ and protects from ALD.^7^ Small-molecules, such as SCFAs (acetate, propionate, butyrate) and Trp metabolites (indole metabolites and others), synthesized by intestinal bacteria, are secreted into the intestinal lumen and act as endogenous agonists of Ahr.^8^^,^^21^^,^^22^ Intestinal dysbiosis due to alcohol consumption results in impaired bacterial metabolism, including Trp metabolism^7^^,^^34^ and SCFAs synthesis.^35^ As a result, there is reduced Ahr signaling, loss of TJs, impairment of intestinal barrier function, and worsening of ALD.^7^^,^^18^ The present study highlights that overexpression of OPN in IECs or oral administration of mOPN prevents the alcohol-mediated downregulation of Ahr signaling in IECs. Previous studies demonstrated that alcohol consumption lowers the abundance of bacteria associated with Trp metabolism and synthesis of SCFAs.^7^^,^^35^ We show that OPN stimulates growth of Trp-metabolizing and SCFAs-synthesizing bacteria in the intestine under alcohol feeding. These include *Bifidobacterium*,^23^^,^^36^^,^^37^ *Eubacterium*,^23^^,^^37^ *Prevotella*,^38^^,^^39^ *Allloprevotella*,^40^ *Desulfovibrio*,^36^^,^^41^ *Butyricicoccus*,^42^ *Butyricimonas*,^43^ and *Roseburia*.^23^^,^^44^^,^^45^ An increase in these bacteria enhances synthesis of SCFAs from undigested carbohydrates and indole derivatives from Trp catabolism in the gut. This is revealed by high levels of SCFAs (acetate, propionate, butyrate) and indole compounds (3-indoleacrylic acid, 3-indoleacrylic acid, 3-indolepropionic acid, 3-indoxyl sulfate, indole) in portal serum from *Opn*^KI IEC^ mice and from WT mice with oral administration of mOPN. These SCFAs and indole derivatives are well-known ligands for Ahr that activate it in IECs. SCFA and indole signaling through Ahr in IECs, increases the expression of TJ proteins, as shown by increased mRNA expression of *Occludin*, *JamA*, and *Jam4* in IECs from jejunum of *Opn*^KI IEC^ and *Claudin-3* and *JamA* in IECs from jejunum of WT mice with oral administration of mOPN. This preserves the gut barrier function and limits translocation of bacteria and bacterial products from the gut to the portal blood.

In ALD, disruption of the intestinal epithelial barrier enhances intestinal permeability^1^ and allows translocation of bacteria and bacterial products from the gut lumen to the portal circulation.^2^ Upon reaching the liver, they stimulate KCs, infiltrating MFs, and neutrophils, to produce proinflammatory cytokines, which damage hepatocytes.^3^ We demonstrate that OPN preserves the gut barrier function and blocks translocation of bacterial products from the gut to the liver as seen by reduced portal serum LPS and RNA expression of *Tnfα*, interleukin-1β *(Il1β)*, *Ccl2*, and *Ccl3* in the liver from ethanol-fed *Opn*^KI IEC^ mice and ethanol-fed WT mice with oral administration of mOPN.

Additionally, we demonstrate comparable alcohol-induced liver injury between male and female mice; this could be because of the genetic background of the mice and/or the ethanol feeding model. However, ethanol-fed WT and *Opn*^KI IEC^ females have higher liver TGs than ethanol-fed males. Ethanol-fed WT females have higher *Il1β* expression in liver, whereas ethanol-fed WT and *Opn*^ΔIEC^ males show increased gut permeability and higher *Tnfα*, *Ccl2*, and *Ccl3* mRNA expression in liver than ethanol-fed females. Further, based on liver TG and serum ALT activity, the protective effects of OPN overexpression are greater in males than in females.

Our study has some limitations. First, the two mouse models, *Opn*^KI IEC^ and *Opn*^ΔIEC^, show heterogeneity in the extent of alcohol-induced liver injury. Overexpression of OPN in IECs prevents the alcohol-induced increase in gut permeability, and deletion of OPN from IEC increases gut permeability after alcohol exposure but does not show further worsening. Second, deletion of OPN from IECs does not downregulate the expression of AMPs, *Ahr*, and TJs in IECs from jejunum. Third, FMT from ethanol-fed WT to OPN ^KI IEC^ and WT mice lacks the OPN ^KI IEC^ and WT mice without FMT as control. Last, in the FMT experiment, only male mice were used; therefore, these results may be gender-specific.

In conclusion, we show that natural induction plus overexpression of OPN in IECs, or oral administration of mOPN during ethanol feeding, protect from ALD by preserving the gut-liver axis. OPN increases AMPs such as *Reg3β* and *Reg3γ* in IECs, which regulate the IM and prevent alcohol-mediated loss of Trp-metabolizing and SCFAs-synthesizing bacteria in the gut. This increases Ahr signaling in IECs, prevents disruption of the intestinal epithelial barrier, and protects from ALD. Therefore, induction of OPN in the intestine or oral administration of mOPN could benefit patients with ALD. The latter therapeutic approach is feasible, as administration of OPN has proven safe, for example, incorporated in infant formulas as Lacprodan OPN-10 (Arla Foods) or as bovine mOPN in clinical trials to study its effect on growth, health, and immune function in infants (NCT04306263, NCT03331276, NCT00970398).

## Materials and Methods

### General Methodology

Details on general methodology, such as immunohistochemistry for OPN, H&E staining, measurement of serum ALT activity, liver TGs, serum alcohol, total RNA extraction, quantitative real-time polymerase chain reaction analysis, and measurement of FITC-dextran and LPS in portal serum are described in previous publications.^2^^,^^46^, ^47^, ^48^, ^49^, ^50^ The sequence of primers used is listed in Table 1. Cytosolic and nuclear proteins were isolated with the compartmental protein extraction kit (Millipore, Burlington, MA). Western blot analysis of STAT3, pSTAT3, Histone H3, AHR, REG3G, and GAPDH was performed using antibodies 9132S, 9145S, and 9715S (Cell Signaling Technology, Inc, Danvers, MA), MA1-514 and PA5-50450 (Invitrogen, Carlsbad, CA), and sc-32233 (Santa Cruz, Dallas, TX), respectively.

**Table 1:** Sequence of Primer Pairs Used in RT-PCR Analysis

Genes	Forward primers (5'-3')	Reverse primers (5'-3')
*16S*	AGAGTTTGATCCTGGCTCAG	TGCTGCCTCCCGTAGGAGT
*18S*	CGCGGTTCTATTTTGTTGGT	AGTCGGCATCGTTTATGGTC
*Ahr*	TGACAGAAATGGAGGCCAGGA	CTGCTGTGACAACCAGCACAAA
*β-actin*	AGCCATGTACGTAGCCATCC	CTCTCAGCTGTGGTGGTGAA
*Ccl2*	GGCATCCCTCTACCCAAGAC	GGGCGTTAACTGCATCTGGA
*Ccl3*	TTCTCTGTACCATGACACTCTGC	CGTGGAATCTTCCGGCTGTAG
*Claudin3*	TTCCCAAGAACTGGGCTGGG	GCCCGTTTCATGGTTTGCCT
*Gapdh*	AGCGAGGAACAGCGACAGAA	ACTCCCATGGTTGGGCTCTG
*Il1β*	TGCCACCTTTTGACAGTGATG	TGATGTGCTGCTGCGAGATT
*Il33*	GGCTCACTGCAGGAAAGTACAG	TTGCCGGGGAAATCTTGGAG
*Jam4*	AAACGCAGCAGTAGCCTTCC	TGAACCTTGGACCTCTCAGCA
*JamA*	TAATGGGCACCGAGGGGAAA	GAGCAGTGTACACCGAACCCT
*Occludin*	TTGAAAGTCCACCTCCTTACAGA	CCGGATAAAAAGAGTACGCTGG
*Opn*	GCAGTCTTCTGCGGCAGGCA	GGGTCAGGCACCAGCCATGTG
*Reg3β*	AACAGCCTGCTCCGTCATGT	AACTAATGCGTGCGGAGGGT
*Reg3γ*	CGCTGAAGCTTCCTTCCTGT	CCCATCCACCTCTGTTGGGTT
*Tnfα*	CTGAACTTCGGGGTGATCGG	GGCTTGTCACTCGAATTTTGAGA

qRT-PCR, Quantitative real-time polymerase chain reaction.

### Mice

**Figure 9:**
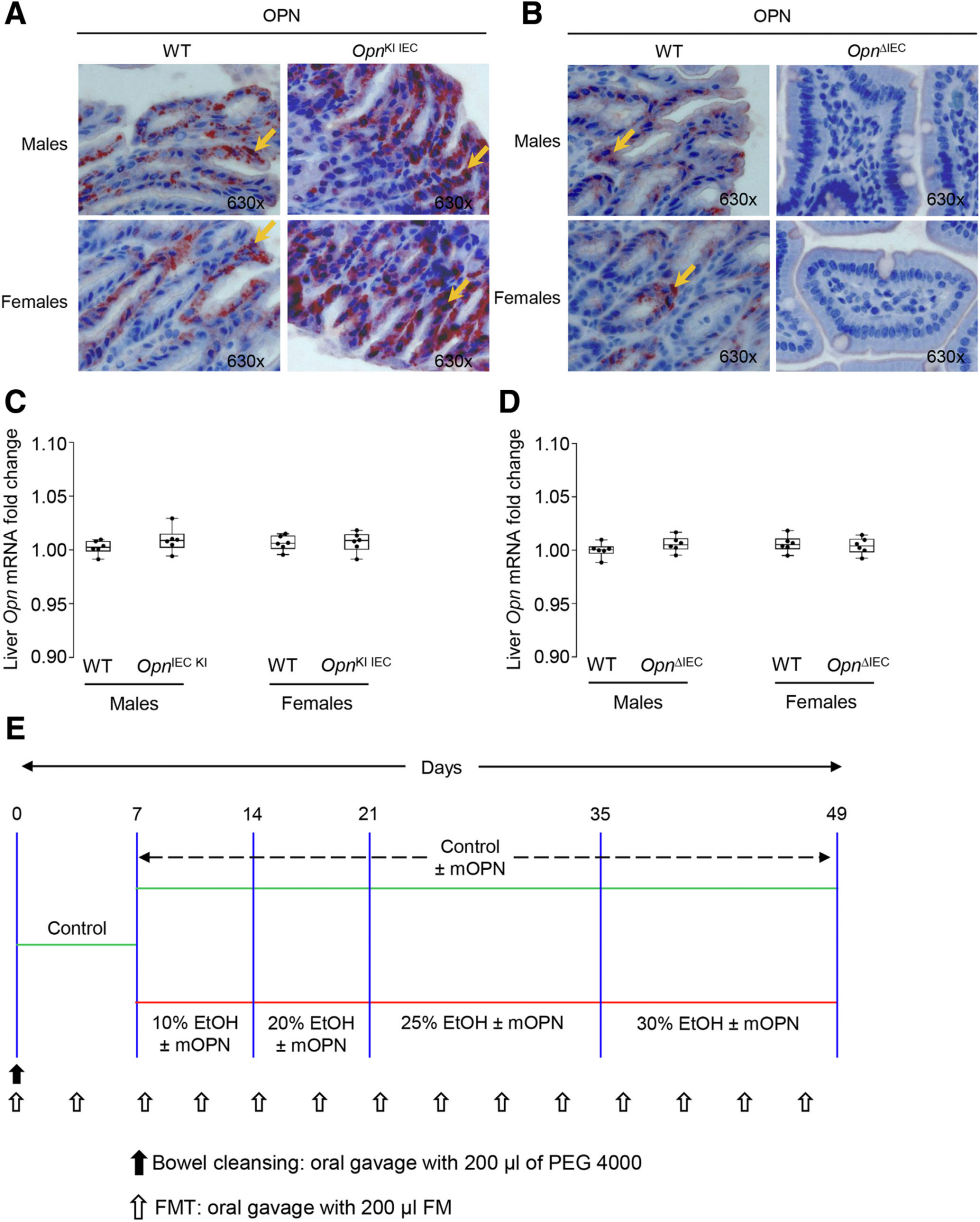
**Validation of the targeting strategy and experimental design of the FMT.** OPN immunohistochemistry in the jejunum from male and female mice fed control diet (*orange arrows* indicate OPN staining in IECs) (*A–B*). *Opn* mRNA expression in liver from male and female *Opn*^KI IEC^ and *Opn*^ΔIEC^ mice fed control diet. Data were normalized with *Gapdh* as housekeeping gene, and FC was calculated against WT (n = 6/group) (*C–D*). Experimental design of the FMT (*E*).

*Opn*^*fl/fl*^ mice were generated in our laboratory. The *Opn*^*loxP*^ allele was created inserting *loxP* sites to remove exons 4–7. *Opn*-Stop^*fl/fl*^ mice were donated by Dr Panoutsakopoulou (Biomedical Research Foundation, Academy of Athens, Greece).^51^ Both *Opn*-Stop^*fl/fl*^ and *Opn*^*fl/fl*^ mice were bred with *Vili-*Cre (JAX004586, the Jackson Laboratory, Bar Harbor, ME) to generate IEC-specific knock-in (*Opn*^KI IEC^) and knock-out (*Opn*^ΔIEC^) mice, respectively. *Villi-Cre* mice were used as controls and referred to as WT on the text or figures for simplicity purposes only. The targeting strategy was validated by immunohistochemistry in the jejunum (Figure 9, *A–B*). We also confirmed that overexpression or deletion OPN in IECs does not affect OPN expression in the liver (Figure 9, *C–D*). All mice were in C57BL/6J background and lacked a liver or intestine phenotype in the absence of treatment.

### Model of Alcohol-induced Liver Injury

The LDC model was used to provoke early alcohol-induced liver injury.^52^ The control and ethanol LDC diets (Bio-Serv Inc, Frenchtown, NJ) are equicaloric and have the same composition of fat (42% of calories) and protein (16% of calories). The content of carbohydrates is 42% of total calories (dextrin-maltose) in the control diet and 12% of total calories in the ethanol diet, where up to 30% of carbohydrate-derived calories are replaced by ethanol.^52^ Equal number of male and female mice (12 weeks old) were acclimatized to the liquid diet by feeding control diet for 7 days. Then, the percentage of ethanol-derived calories was progressively increased from 10% (1 week) to 20% (1 week), 25% (2 weeks), and 30% (2 weeks) in the ethanol group. The control groups remained on control diet. Mice were gavaged with 4 μL/g of body weight of FITC-dextran 4 hours before sacrifice. Liver and body weight were recorded upon sacrifice to calculate the liver-to-body weight ratio. Systemic and portal blood were drawn under anesthesia from the submandibular and portal veins, respectively, to obtain serum. Serum, liver, intestine and stool samples were collected and stored at −80 °C for further analysis.

### Pathology

Sections from left liver lobe, duodenum, jejunum, ileum, and colon were obtained, fixed in 10% neutral formalin buffer, processed, and sectioned (4 μm) for H&E staining. Liver injury was determined by a liver pathologist according to the Brunt classification.^53^ The steatosis grade was 0 = <5%; 1 = 5% to 33%; 2 = >33% to 66%; and 3 = >66%. Steatosis was noted to be macrovascular, microvascular, or both. Inflammation was noted based on the presence of inflammatory foci and was scored as follows: 0 for none; 1 for <2 foci/field; 2 for 2 to 4 foci/field; and 3 for >4 foci/field. Ten fields per section were observed to for semiquantitative scoring of liver steatosis and inflammation.

### FMT

Three independent FMT experiments were performed in 12-week-old male mice. First, FMT from *Opn*^KI IEC^ to WT (C57BL/6J, Stock No. 000664) mice. Second, FMT from WT fed ethanol for 6 weeks to *Opn*^KI IEC^ mice. Third, FMT from WT fed ethanol for 6 weeks to WT mice. Briefly, feces (100 mg) were dissociated in 1 mL of sterile saline solution by vigorous shaking followed by centrifugation at 800 × *g* for 3 minutes.^54^ The supernatant containing the FM suspension was stored in aliquots (200 μL) at −80 °C until FMTs were performed. Mice were fasted for 1 hour, and the bowel was cleansed with polyethylene glycol (Macrogol 4000, Thermo Fisher Scientific, Carlsbad, CA). Polyethylene glycol (200 μL of a solution of 425 g/L in water) was administered by oral gavage.^7^ After 4 hours, mice received 200 μL of the FM suspension by oral gavage. Mice had free access to control or ethanol diet for 6 weeks. In the third FMT experiment, mice received control or ethanol diet supplemented with purified bovine mOPN (gift from Arla Foods, Viby, Denmark) at a concentration of 200 μg/mL.^1^ FMT was performed twice a week for 6 weeks, and bowel cleansing was done once before the first FMT^7^ (Figure 9, *E*).

### Metagenomics

DNA was isolated from fecal samples using the DNeasy power soil kit (Qiagen, Germantown, MD).^55^ Sequencing of 16S rRNA was performed at the Argonne National Laboratory (Lemont, IL) using an Illumina micro MiSeq platform.^56^^,^^57^ The V4 region of the 16S rRNA gene was amplified using specific barcodes and primer sequences listed in Table 2. Data were analyzed using the Illumina 16S metagenomics App in BaseSpace. Demultiplexed fastq sequences were processed for quantitative and qualitative insight into the microbial ecology. Alpha and beta diversity were determined based on the Shannon’s index and weighted UniFrac distances, respectively. For comparisons, nonparametric 1-way analysis of variance followed by the Fisher LSD post-hoc tests or a combination of fold change analysis and *t* tests (volcano plot), were used, and a cutoff of ≤ .05 adjusted *P*-value (false discovery rate) or *P*-value was considered for the statistical significance.

**Table 2:** Sequence of Barcodes and Primers Used in 16S rRNA Sequencing

SI.No	Barcode sequence	Linker primer sequence
S1	GTCCGCAAGTTA	GTGTGYCAGCMGCCGCGGTAA
S2	CAACACATGCTG	GTGTGYCAGCMGCCGCGGTAA
S3	CATACCGTGAGT	GTGTGYCAGCMGCCGCGGTAA
S4	GTCCATGGTTCG	GTGTGYCAGCMGCCGCGGTAA
S5	ACCATTACCATT	GTGTGYCAGCMGCCGCGGTAA
S6	TGGTAAGAGTCT	GTGTGYCAGCMGCCGCGGTAA
S7	CCAGCCTTCAGA	GTGTGYCAGCMGCCGCGGTAA
S8	ATTCAGATGGCA	GTGTGYCAGCMGCCGCGGTAA
S9	TTATTCTCTAGG	GTGTGYCAGCMGCCGCGGTAA
S10	TTCGTGAGGATA	GTGTGYCAGCMGCCGCGGTAA
S11	GCGTCATGCATC	GTGTGYCAGCMGCCGCGGTAA
S12	CCTCGGGTACTA	GTGTGYCAGCMGCCGCGGTAA
S13	CTACTAGCGGTA	GTGTGYCAGCMGCCGCGGTAA
S14	CGATTTAGGCCA	GTGTGYCAGCMGCCGCGGTAA
S15	GCTTGGTAGGTT	GTGTGYCAGCMGCCGCGGTAA
S16	AGGCGCTCTCCT	GTGTGYCAGCMGCCGCGGTAA
S17	ACCTGATCCGCA	GTGTGYCAGCMGCCGCGGTAA
S18	GAGATTTAAGCA	GTGTGYCAGCMGCCGCGGTAA
S19	TGGGTCCCACAT	GTGTGYCAGCMGCCGCGGTAA
S20	ATTCTGCCGAAG	GTGTGYCAGCMGCCGCGGTAA
S21	TTGCCTGGGTCA	GTGTGYCAGCMGCCGCGGTAA
S22	TCGTAAGCCGTC	GTGTGYCAGCMGCCGCGGTAA
S23	ATTAGATTGGAG	GTGTGYCAGCMGCCGCGGTAA
S24	TTAGCCCAGCGT	GTGTGYCAGCMGCCGCGGTAA
S25	ACTAGGATCAGT	GTGTGYCAGCMGCCGCGGTAA
S26	TACACCTTACCT	GTGTGYCAGCMGCCGCGGTAA
S27	AGTGTCGATTCG	GTGTGYCAGCMGCCGCGGTAA
S28	ATCTCGCTGGGT	GTGTGYCAGCMGCCGCGGTAA
S29	ATTCCATTTAGA	GTGTGYCAGCMGCCGCGGTAA
S30	CTGCTGGGAAGG	GTGTGYCAGCMGCCGCGGTAA
S31	CAGGGAGGATCC	GTGTGYCAGCMGCCGCGGTAA
S32	GGACTATCGTTG	GTGTGYCAGCMGCCGCGGTAA
S33	CAATTCTGCTTC	GTGTGYCAGCMGCCGCGGTAA
S34	GTTATACATTCA	GTGTGYCAGCMGCCGCGGTAA
S35	GATGTCATAGCC	GTGTGYCAGCMGCCGCGGTAA
S36	CGTGACAATAGT	GTGTGYCAGCMGCCGCGGTAA
S37	GAGGGCGTGATC	GTGTGYCAGCMGCCGCGGTAA
S38	ATGGTCACAAAC	GTGTGYCAGCMGCCGCGGTAA
S39	TTGTATGACAGG	GTGTGYCAGCMGCCGCGGTAA
S40	TGGAAACCATTG	GTGTGYCAGCMGCCGCGGTAA
S41	AACCCTAACTGG	GTGTGYCAGCMGCCGCGGTAA
S42	TATAACAATCTC	GTGTGYCAGCMGCCGCGGTAA
S43	ACGAGGAGTCGA	GTGTGYCAGCMGCCGCGGTAA
S44	TGGCGATACGTT	GTGTGYCAGCMGCCGCGGTAA
S45	CTTTCAGGACCG	GTGTGYCAGCMGCCGCGGTAA
S46	CGATCACCACAA	GTGTGYCAGCMGCCGCGGTAA
S47	AGTAGGAGGCAC	GTGTGYCAGCMGCCGCGGTAA
S48	TGTACGGATAAC	GTGTGYCAGCMGCCGCGGTAA
S49	GGTTCCATTAGG	GTGTGYCAGCMGCCGCGGTAA
S50	AAGGAAGTATAT	GTGTGYCAGCMGCCGCGGTAA
S51	TTGTTACGTTCC	GTGTGYCAGCMGCCGCGGTAA
S52	CGCTACAACTCG	GTGTGYCAGCMGCCGCGGTAA
S53	ATCAGCCAGCTC	GTGTGYCAGCMGCCGCGGTAA
S54	ATTTCTTAGCCA	GTGTGYCAGCMGCCGCGGTAA
S55	ATTCGCCAAGAA	GTGTGYCAGCMGCCGCGGTAA
S56	AGTGCGTTCTAG	GTGTGYCAGCMGCCGCGGTAA
S57	CAATTAATGTAT	GTGTGYCAGCMGCCGCGGTAA
S58	CTGTGATCGGAT	GTGTGYCAGCMGCCGCGGTAA
S59	GATCGGTTAATG	GTGTGYCAGCMGCCGCGGTAA
S60	TGGACGGCCCAG	GTGTGYCAGCMGCCGCGGTAA

### Measurement of Trp Metabolites and SCFAs

Trp and its metabolites (kynureine and indoles) and SCFAs were measured in portal serum by targeted metabolite profiling at the Metabolomics Core from the Roy J. Carver Biotechnology Center at the University of Illinois at Urbana-Champaign.

Trp and its metabolites were quantified using liquid chromatography–mass spectrometry (LC/MS). Briefly, 30 μL of serum were deproteinized with 70 μL of methanol and centrifuged to obtain the supernatant. The supernatants and the standards for Trp and its metabolites were spiked with 5 μL of the internal standard (DL-Chlorophenyl alanine, 0.01 mg/ml) and quantified by LC/MS in a Vanquish high-performance liquid chromatography (Thermo Fisher Scientific, Waltham, MA) with a Polaris 3 C18-Ether 2x100 mm (3 μ) column at a flow rate of 500 μL/min. As mobile phases, 0.1% formic acid in water (A) and methanol (B) were used with the following gradient: 0 to 0.5 minutes–0% B, 3 minutes–100% B, 3 to 4.5 minutes–100% B, and 4.5 to 6 min–0% B. Mass spectrometry was performed in a TSQ Altis LC-MS/MS (Thermo Fisher Scientific). Data were acquired in both negative and positive SRM modes. Integration and quantitation of the peaks were performed using Thermo TraceFinder. For Trp, 3-Indoleacrylic acid and 3-Indoxyl, a standard curve 200 to 0.5 μg/mL was used, and for the rest of the metabolites, a standard curve of 500 to 0.1 ng/mL was used.

After the LC/MS measurements, the same samples were used to quantify SCFAs by gas chromatography/MS on an Agilent 7890 gas chromatograph, with a 5977A mass selective detector and a 7693 autosampler (Agilent Inc, Palo Alto, CA). Using a split mode (15:1), 1 μL of sample was injected and analyzed on a 30-m HP-INNOWAX column with 0.25 mm inner diameter and 0.25 μm film thickness (Agilent). An injection temperature of 200 °C, mass selective detector transfer line of 200 °C, adjusted ion source of 230 °C, and a constant flow rate of 1 mL/min of helium carrier gas were used for the analysis. The temperature program was 2 minutes at 70 °C, followed by an oven temperature ramp of 10 °C/minute to 190 °C and 40 °C to 240 °C for a final 2 minutes. The MS was performed in positive electron impact mode at 69.9 eV ionization energy in m/z 30 to 300 scan range in combined scan and selected ion monitoring modes targeted for m/z 43, 45, 46, 60, and 74. Using Mass Hunter Quantitative Analysis B.08.00 (Agilent) software, targeted peaks were evaluated. To generate standard curves, 100 to 0.01 mg/L concentrations were used.

### Isolation of IECs

IECs were isolated from the jejunum. In this protocol, first, the adhesions between the IECs were disrupted by chelating Ca^+2^ ions, and then, mechanical separation of IECs was performed using a vortex.^58^

### Study Approval

All mice received humane care according to the criteria outlined in the guide for the care and use of laboratory animals prepared by the National Academy of Sciences and published by the National Institutes of Health. Housing and husbandry conditions were approved by the Institutional Animal Care and Use Committee office at the University of Illinois at Chicago prior to initiation of the studies. All in vivo experiments were carried out according to the Animal Research: Reporting of In Vivo Experiments (ARRIVE) guidelines.

### Statistical Analysis

Statistical analyses were performed using GraphPad Prism v7 with statistical significance set at *P* < .05. Comparisons between 2 groups were performed using the nonparametric *t* tests (Mann-Whitney *U* test). For comparisons between 3 groups, nonparametric 1-way analysis of variance (Kruskal-Wallis test) followed by post hoc comparisons by the Bonferroni method were used.
